# Efficacy of Appropriate Techniques of Traditional Chinese Medicine and Physical Therapy in Patients With Insulin‐Associated Lipohypertrophy: A Systematic Review and Meta‐Analysis

**DOI:** 10.1155/ije/5589949

**Published:** 2026-08-27

**Authors:** Liqing Jiang, Honglian Fang, Xumei Tao, Liping Wang

**Affiliations:** ^1^ Department of Gerontology, The Affiliated Nanjing Brain Hospital of Nanjing Medical University, Nanjing, China, njmu.edu.cn

**Keywords:** HbA1c, lipohypertrophy, meta-analysis, physical therapy, Traditional Chinese Medicine

## Abstract

**Objectives:**

The study aimed to investigate the effectiveness of Traditional Chinese Medicine (TCM) and physical therapy on the lipohypertrophy (LH) people by conducting meta‐analyses of randomized controlled trials (RCTs).

**Methods:**

We performed a literature search on China Knowledge Network Infrastructure (CNKI), Wanfang Data, VIP.com, PubMed, Web of Science, Cochrane Library, and ClinicalTrials.gov in June 30, 2024, for RCTs. Standardized mean difference (SMD) or odds ratio (OR) with 95% confidence intervals (CIs) was calculated. Subgroup analyses, sensitivity analyses, and publication bias analyses were also conducted.

**Results:**

A total of 31 RCTs involving 3496 participants with LH were included in this analysis. Meta‐analyses revealed that appropriate TCM interventions significantly improved the curative effect in individuals with LH (OR = 5.52; 95% CI: 3.98–7.65; *p* < 0.0001). Furthermore, preliminary analyses suggested potential benefits of the TCM intervention on HbA1c levels (SMD = −0.42; 95% CI: −0.63 to −0.22; *p* < 0.0001), as well as on fasting blood glucose (FBG) and 2‐h postload plasma glucose levels.

**Conclusion:**

The present meta‐analysis suggests that appropriate technology of TCM intervention was effective in improving the curative effect, HbA1c level, FBG, and 2hPG. Physical therapy may be effective for the treatment of LH. However, the quality of the included RCTs for appropriate technology of TCM is low to moderate and the number of RCTs for physical therapy is limited.

## 1. Introduction

According to the Global Diabetes Data Report, 537 million adults (20–79 years) are living with diabetes. This number is predicted to rise to 643 million by 2030 and 783 million by 2045. Diabetes caused at least USD 966 billion dollars in health expenditure—a 316% increase over the last 15 years [1]. Insulin therapy is the most important treatment approach of diabetes mellitus, including insulin pen and insulin pump therapy [2]. Lipohypertrophy (LH) is the most common cutaneous alteration and skin‐related adverse effect of insulin therapy, which may depend on the adipogenic effect of the hormone and other contributing factors, including injection technique, needle size, frequency of injections at the same site, and duration of insulin use, resulting in thickening of subcutaneous tissues and the formation of visible and/or invisible, palpable, and/or nonpalpable masses [3]. About 38.0%–76.3% of diabetic people present with LH [2, 4].

Studies have shown that LH affects insulin uptake, makes glycemic control more difficult, places an additional financial burden on society and families, and increases the risk of various complications of diabetes, especially unexplained hypoglycemia [5]. In addition, some LH can cause pain, inflammation, and rupture, thus increasing people’s discomfort and affecting their quality of life (QoL) [6].

The mechanisms of LH occurrence and development are unclear, and various hypotheses have been proposed for the treatment of LH [7]. LH is often treated with preventive measures, such as changing needles and injection sites, and choosing appropriate needles [8, 9]. A meta‐analysis of the efficacy of injection technique in people with LH found that injection technique may slightly reduce the total daily dose of insulin. However, the same meta‐analysis included only two studies with considerable methodological heterogeneity—employing variable diagnostic criteria (palpation, visual inspection, or ultrasound) and differing in injection frequency and needle size—which precluded definitive conclusions regarding its effects on HbA1c, hypoglycemia occurrence, and LH resolution [10]. Indeed, rigorously designed studies by Gentile and colleagues have demonstrated that proper injection technique education can yield significant improvements in metabolic variables and hypoglycemia rates [8, 9]. Nevertheless, the delayed therapeutic response associated with education‐based interventions may pose challenges to patient adherence over time [10], which highlights the need for adjunctive therapeutic strategies that offer more immediate and observable effects to complement education‐based approaches and potentially improve overall compliance and outcomes.

Previous case reports have found that surgical resection and liposuction are effective in relieving pain, reducing insulin injections, and lowering the incidence of diabetes‐related complications [11, 12]. In addition, it has been found that TCM appropriate techniques and physical therapy may promote shrinkage and/or cure of LH and may improve HbA1c levels and/or fasting blood glucose (FBG) levels and/or postprandial 2‐h blood glucose (2hPG) levels and/or LH severity.

Therefore, this study is a comprehensive collection of randomized controlled trials (RCTs) of noninjection technique intervention protocols with the aim of conducting a systematic review and meta‐analysis in order to determine the effects of noninjection technique interventions on HbA1c, FBG, and postprandial 2hPG with LH.

## 2. Methods

This systematic review is based on the recommendations of the Initiative Report on Systematic Reviews and Meta‐Analyses (PRISMA) [13]. PRISMA checklist can be found in Supporting Information 1. The study protocol was registered with the International Prospective Systems Evaluation Register (PROSPERO; Registration No. CRD42024526734).

### 2.1. Search Strategy

The search time was from the establishment of the database to April 30, 2024. Chinese databases that were searched included China Knowledge Network Infrastructure (CNKI), Wanfang Data Knowledge Service Platform, and VIP.com (VIP). English databases that were searched included PubMed, Web of Science, and the Cochrane Library. Search terms included the technology of Traditional Chinese Medicine (TCM), photon therapy, diabetes, and random. The search strategies are presented in online Supporting Information 2. We checked the reference lists of the studies, including international guidelines, and identified the reference lists of eligible studies and articles citing eligible studies. For each excluded study, the reasons for exclusion were documented. In cases where the two independent reviewers disagreed on the eligibility of a study, a third researcher was invited to review the study and make the final decision. The reasons for exclusion provided by the third researcher were also recorded.

### 2.2. Study Selection and Exclusion Criteria

Studies were selected if they met the following inclusion criteria: (1) Participants: (1) meeting the World Health Organization’s diagnostic criteria for Type 2 or Type 1 diabetes mellitus; (2) LH was diagnosed using either method, consistent with the internationally recommended palpation techniques described by Gentile and the FITTER recommendations, and was characterized by at least one LH measuring ≥ 5 mm in diameter [14–16]; and (3) voluntary participation and signing of informed consent. (2) Intervention: noninjection technology intervention. (3) Comparator: education on different concentrations of magnesium sulfate moist heat packs and insulin injection techniques. (4) Outcomes: curative effect, HbA1c, FBG, and 2hPG. (5) Study design—RCTs. (6) Publication type: ability to obtain full text and complete data.

Exclusion criteria were as follows: (1) duplicate publication; (2) ambiguous endpoint indicators or inability to merge, transform, and use data in the original study; (3) unavailability of full text; and (4) stepped wedge cluster RCTs.

### 2.3. Data Extraction

According to the abovementioned electronic database search strategy, two independent researchers searched the Chinese and English electronic databases, respectively. The details of extracted data are shown in Table 1, including author, year, country, sample size, age, sex, estimated diabetes duration, insulin treatment duration, intervention types, intervention details, detection method of LH, and outcome indicators.

**TABLE 1:** Characteristics and summary findings of included studies.

Study	Sample size	Subject characteristics	Estimated diabetes duration (years)	Insulin treatment duration (years)	Intervention types	Intervention details	Detection method of LH	Reported outcome
Ai 2013 [17]	40 E: 20 C: 20	Age (years): 54.6 (E), 54.4 (C) Sex: not mentioned	E: 8.7 C: 8.6	E: 5.2 C: 5.2	Appropriate technology of Traditional Chinese Medicine and physical therapy	E: warm compress of traditional Chinese medicine for 30 min twice a day for a month C: 33% magnesium sulfate external application and infrared irradiation for 30 min twice a day for a month	Visible and/or palpable LH determined by healthcare	(1) Curative effect

Chen 2017 [18]	97 E: 51 C: 46	Age (years): 66. 3 ± 3. 9 (E), 66. 6 ± 4. 1 (C) Sex: 55 (M), 42 (F)	E: 7.2 ± 3.1 C: 7.9 ± 3.5	Not mentioned	Appropriate technology of Traditional Chinese Medicine	E: Yunnan Baiyao combined with alcohol patch, change every 24 h, a total of 2 weeks C: Apply 50% magnesium sulfate topically twice a day for 2 weeks	Visible and/or palpable LH determined by healthcare	(1) Curative effect (2) HbA1c

Wu 2017 [19]	56 E: 28 C: 28	Age (years): 65 (E), 68 (C) Sex: 30 (M), 26 (F)	Not mentioned	Not mentioned	Appropriate technology of Traditional Chinese Medicine	E: TCM Tongyu powder was applied externally for 12 h for 12 days s C: 50% magnesium sulfate wet hot compress twice a day for 12 days	Visible and/or palpable LH determined by healthcare	(1) Curative effect

Xiong 2017 [20]	46 E: 23 C: 23	Age (years): 55.3 (E), 54.6 (C) Sex: 24 (M), 22 (F)	E: 8.9 C: 8.7	E: 5.6 C: 5.6	Appropriate technology of Traditional Chinese Medicine and Physical therapy	E: Photon therapy instrument combined with traditional Chinese medicine Tongyu powder external application for 30 min, twice a day, a total of 1 month C: Photon therapy device combined with 50% magnesium sulfate external application for 30 min, twice a day, a total of 1 month	Visible and/or palpable LH determined by healthcare	(1) Curative effect

Gu 2018 [21]	60 E: 30 C: 30	Age (years): 32–76 (E), 31–75 (C) Sex: not mentioned	E: 4–18 C: 5–16	E: 1–9 C: 11 months −8	Appropriate technology of Traditional Chinese Medicine	E: External application of Sihuang paste, once a day, for 7 days C: 50% magnesium sulfate wet compress, 3 times a day for 7 days	Visible and/or palpable LH determined by healthcare	(1) Curative effect

Hu 2018 [22]	30 E: 15 C: 15	Age (years): 35–72 (E), 43–78 (C) Sex: 17 (M), 13 (F)	Not mentioned	Not mentioned	Appropriate technology of Traditional Chinese Medicine	E: Apply Chinese medicine for no less than 4 h each time, once a day, for 15 days C: 50% magnesium sulfate wet compress, once a day, 1 h each time, a total of 15 days	Visible and/or palpable LH determined by healthcare	(1) Curative effect

Min 2018 [23]	48 E: 24 C: 24	Age (years): 69.4 ± 2.4 (E), 68.5 ± 3.5 (C) Sex: 27 (M), 21 (F)	Not mentioned	Not mentioned	Appropriate technology of Traditional Chinese Medicine	E: External application of ichthyosis fat paste, twice a day, 30 min of hot compress, a total of 1 month C: 33% magnesium sulfate wet compress, once a day, 1 h each time, a total of 1 month	Visible and/or palpable LH determined by healthcare	(1) Curative effect

Wang Lina 2018 [24]	84 E: 42 C: 42	Age (years): 61.21 ± 7.47 (E), 59.79 ± 10.47 (C) Sex: 37 (M), 54 (F)	Not mentioned	Not mentioned	Appropriate technology of Traditional Chinese Medicine	E: 50% magnesium sulfate. Magnesium sulfate combined with egg white wet compress, change once every 6–8 h, change 3 to 4 times a day, a total of 7 days C: 50% magnesium sulfate wet compress, once a day, change once every 20–30 min, a total of 7 days	Visible and/or palpable LH determined by healthcare	(1) Curative effect

Wu 2018 [25]	68 E: 34 C: 34	Age (years): 48.2 ± 4.5 (E), 48.7 ± 3.8 (C) Sex: 47 (M), 37 (F)	E: 4.5 C: 5.0	E: < 5: 17; 5–15: 12; ≥ 15: 5 C: < 5: 15; 5–15: 18; ≥ 15: 1	Appropriate technology of Traditional Chinese Medicine	E: Moxibustion therapy, once a day for 10 min for 14 days C: 25% magnesium sulfate wet compress, twice a day, 15 min each time, a total of 14 days	Visible and/or palpable LH determined by healthcare	(1) Curative effect (2) LH diameter

Wang Jun 2018 [26]	91 E: 46 C: 45	Age (years): 59.09 ± 11.99 (E), 61.44 ± 6.87 (C) Sex: 32 (M), 36 (F)	E: 2–5: 13; 6–10: 14; 11–15: 13; 16–20: 5; > 20: 1 C: 2–5: 13; 6–10: 12; 11–15: 12; 16–20: 6; > 20: 2	Not mentioned	Appropriate technology of Traditional Chinese Medicine	E: Moxa box moxibustion, once a day, 15 min each time, a total of 14 days C: education on injection technique	Visible and/or palpable LH determined by healthcare	(1) Curative effect (2) HbA1c (3) FBG (4) 2hPG

Guo 2019 [27]	80 E: 40 C: 40	Age (years): not mentioned Sex: not mentioned	Not mentioned	Not mentioned	Appropriate technology of Traditional Chinese Medicine	E: Comfrey oil patch, 30 min twice a day for 5 days C: 50% magnesium sulfate wet compress, twice a day, 30 min each time, a total of 5 days	Visible and/or palpable LH determined by healthcare	(1) Curative effect

Luo 2019 [28]	100 E: 50 C: 50	Age (years): 62.88 ± 5.21 (E), 61.44 ± 2.88 (C) Sex: 48 (M), 52 (F)	E: 12.42 ± 5.85 C: 11.83 ± 3.47	E: 6.11 ± 3.61 C: 5.79 ± 3.19	Appropriate technology of Traditional Chinese Medicine	E: Shuangbai powder was applied externally once a day for 15–30 min each time for 1 month C: education on injection technique	Visible and/or palpable LH determined by healthcare	(1) Curative effect

Song 2019 [29]	78 E: 38 C: 40	Age (years): 57.5 ± 15.9 (E), 54.7 ± 15.8 (C) Sex: 37 (M), 41 (F)	E: 11.00 (6.00,25.00) C: 8.00 (3.00,12.00)	Not mentioned	Appropriate technology of Traditional Chinese Medicine	E: Education on injection technique combined with potato chips for external application, 20–30 min twice a day for 3 months C: Education on injection technique	ultrasound examination	(1) curative effect (2) HbA1c (3) FBG

Ai 2020 [30]	80 E: 40 C: 40	Age (years): 55.8 ± 1.9 (E), 56.7 ± 2.1 (C) Sex: 38 (M), 42 (F)	Not mentioned	E: 6.7 ± 0.3 C: 6.5 ± 0.9	Appropriate technology of Traditional Chinese Medicine and physical therapy	E: TDP combined with tincture wet compress, 30 min each time, once a day, a total of 1 month C: TDP combined with magnesium sulfate wet hot compress, 30 min each time, once a day, a total of 1 month	Visible and/or palpable LH determined by healthcare	(1) LH reduced to less than 50% of the time (2) Time for redness to disappear (3) Time for pain to disappear

Liu 2020 [31]	82 E: 41 C: 41	Age (years): 31.4 ± 3.2 (E), 30.2 ± 3.6 (C) Sex: 46 (M), 36 (F)	E: 6.16 ± 0.67 C: 6.02 ± 0.52	Not mentioned	Appropriate technology of Traditional Chinese Medicine	E: Health education and 50% magnesium sulfate wet hot compress combined with warm moxibustion stick, 15 min each time, a total of 8 weeks C: education on injection technique combined with 50% magnesium sulfate wet hot compress, 15–20 min each time, a total of 8 weeks	Visible and/or palpable LH determined by healthcare	(1) Curative effect

Sha 2020 [32]	146 E: 73 C: 73	Age (years): 31.4 ± 3.2 (E), 30.2 ± 3.6 (C) Sex: 46 (M), 36 (F)	not mentioned	E: < 1:19; 1–3: 26; 4–6: 21; > 6: 7 C: < 1:17; 1–3: 25; 4–6: 23; > 6: 8	Appropriate technology of Traditional Chinese Medicine	E: Traditional Chinese moxibustion combined with ichthyosis ointment: ichthyosis ointment for 30 min each time, 2 times a day, the next day; Traditional Chinese moxibustion therapy for 10–15 min each time, once a day, every other day, a total of 14 days C: 33% magnesium sulfate wet compress, once a day, a hour each time, a total of 14 days	Visible and/or palpable LH determined by healthcare	(1) Curative effect

Xue 2020 [33]	80 E: 40 C: 40	Age (years): 60.59 ± 6.36 (E), 60.31 ± 6.65 (C) Sex: 30 (M), 30 (F)	E: 8.75 ± 4.48 C: 8.59 ± 4.21	E: 5.62 ± 3.72 C: 5.87 ± 3.21	Appropriate technology of Traditional Chinese Medicine	E: Lightning moxibustion for 10 min each time, once a day, a total of 14 days C: 25% magnesium sulfate wet hot compress, change once every 5 min, 15 min each time, twice a day, a total of 14 days	Visible and/or palpable LH determined by healthcare	(1) Curative effect

Shi 2021 [34]	80 E: 40 C: 40	Age (years): 43.18 ± 12.76 (E), 43.54 ± 1.58 (C) Sex: 32 (M), 48 (F)	Not mentioned	Not mentioned	Appropriate technology of Traditional Chinese Medicine	E: Warm patch and mugwort hyperthermia for 20–30 min each time, twice a day, a total of 7 days C: education on injection technique	Visible and/or palpable LH determined by healthcare	(1) Curative effect

Yu 2021 [35]	63 E: 32 C: 31	Age (years): 57.7 ± 10.1 (E), 61.1 ± 8.4 (C) Sex: 27 (M), 36 (F)	E: 8.40 ± 4.27 C: 8.93 ± 4.17	Not mentioned	Appropriate technology of Traditional Chinese Medicine	E: 45 °C hot water hot compress combined with hydrocolloid dressing for 15 min each time, the towel temperature drops when the change, once before going to bed, a total of 14 days C: 45 °C hot water hot compress for 15 min each time, the towel temperature drops when the change, once before going to bed, a total of 14 days	Ultrasound examination	(1) Curative effect

Guo 2022 [36]	120 E: 60 C: 60	Not mentioned	Not mentioned	Not mentioned	Appropriate technology of Traditional Chinese Medicine	E: The comfrey oil patch is changed every 6 h for a total of 5 days C: The potato patch is changed every 6 h for a total of 5 days	Visible and/or palpable LH determined by healthcare	(1) Curative effect

He 2022 [37]	72 E: 36 C: 36	Age (years): 59.11 ± 13.41 (E), 58.68 ± 12.32 (C) Sex: 43 (M), 29 (F)	Not mentioned	E: 6.09 ± 2.45 C: 5.68 ± 3.27	Appropriate technology of Traditional Chinese Medicine	E: External application of Pu Hui powder for at least 6 h each time, once before going to bed, a total of 4 weeks C: education on injection technique	Visible and/or palpable LH determined by healthcare	(1) Curative effect (2) LH diameter

Li 2022 [38]	90 E: 45 C: 45	Age (years): 48.7 ± 10.6 (E), 52.4 ± 12.2 (C) Sex: 31 (M), 39 (F)	Not mentioned	Not mentioned	Appropriate technology of Traditional Chinese Medicine	E: Moxibustion combined with Chinese medicine: local moxibustion once a day, 10 min each time; apply the prepared borneol and wine semen (1g borneol +10 mL 75% alcohol) with a cotton swab on the induration and leave to dry C: education on injection technique	Visible and/or palpable LH determined by healthcare	(1) Curative effect

Su 2022 [39]	120 E: 60 C: 60	Age (years): 56.28 ± 5.26 (E), 57.01 ± 4.72 (C) Sex: 77 (M), 43 (F)	Not mentioned	E: < 5: 21; 5–15: 23; > 15: 16 C: < 5: 22; 5–15: 24; > 15: 14	Appropriate technology of Traditional Chinese Medicine	E: Siko heat ironing 20 min each time, twice a day for 10 days C: 25% magnesium sulfate wet hot compress for 20 min each time, once every 10 min, twice a day for 10 days	Ultrasound examination	(1) Curative effect (2) FBG (3) 2hPG

Wang 2022 [40]	70 E: 35 C: 35	Age (years): 59.00 ± 7.64 (E), 62.57 ± 10.74 (C) Sex: 32 (M), 38 (F)	Not mentioned	Not mentioned	Appropriate technology of Traditional Chinese Medicine	E: Hot compress Pu Huang Bao for 20 min each time, twice a day for 10 days C: 50% magnesium sulfate wet hot compress for 20 min each time, once every 5 min, twice a day for 14 days	Visible and/or palpable LH determined by healthcare	(1) Curative effect

Wang Xiaoyan 2022 [41]	74 E: 37 C: 37	Age (years): 65.42 ± 4.13 (E), 65.39 ± 4.12 (C) Sex: 41 (M), 33 (F)	E: 8.27 ± 1.15 C: 8.25 ± 1.13	Not mentioned	Appropriate technology of Traditional Chinese Medicine	E: Health education combined with moxibustion 15 min each time, once a day C: Health education	Ultrasound examination	(1) Curative Effect (2) LH diameter

Zhou 2022 [42]	40 E: 20 C: 20	Age (years): 55.72 ± 2.37 (E), 54.53 ± 2.28 (C) Sex: 29 (M), 11 (F)	E: 15.28 ± 6.13 C: 15.05 ± 5.77	E: 38.73 ± 14.96 C: 41.05 ± 15.77	Appropriate technology of Traditional Chinese Medicine	E: Anti‐inflammatory pain relief cream 30 min each time, twice a day for 28 days C: 50% magnesium sulfate wet hot compress 30 min each time, twice a day, a total of 28 days	visible and/or palpable LH determined by healthcare	(1) Curative effect (2) HbA1c (3) FBG (4) 2hPG

Xu 2023 [43]	70 E: 35 C: 35	Age (years): 62.57 ± 10.74 (E), 59.00 ± 7.64 (C) Sex: 32 (M), 38 (F)	E: 10.23 ± 5.88 C: 11.02 ± 6.16	Not mentioned	Appropriate technology of Traditional Chinese Medicine	E: Puhuang hot bag wet hot compress, replace once every 5 min, 20 min each time, 2 times a day, a total of 14 days C: 50% magnesium sulfate wet hot compress 20 min each time, twice a day, a total of 14 days	ultrasound examination	(1) FBGc (2) 2hPG

Xu m 2023 [44]	70 E: 35 C: 35	Age (years): 55.35 ± 10.51 (E), 56.77 ± 10.23 (C) Sex: 33 (M), 37 (F)	E: 5.54 ± 2.23 C: 5.32 ± 2.35	Not mentioned				(1) HbA1c (2) FBG (3) 2hPG

Zhao 2023 [45]	90 E: 45 C: 45	Age (years): 63.24 ± 6.24 (E), 64.37 ± 5.98 (C) Sex: 48 (M), 42 (F)	E: 8.27 ± 4.26 C: 9.06 ± 3.57	Not mentioned	Appropriate technology of Traditional Chinese Medicine	E: Lightning moxibustion once a day, 10 min each time, a total of 15 days C: Magnesium sulfate wet hot compress twice a day, 15 min each time, every 5 min to change once, a total of 14 days	Ultrasound examination	(1) Curative effect (2) LH area change

Zhou 2023 [46]	94 E: 47 C: 47	Age (years): 56.61 ± 19.33 (E), 60.32 ± 16.30 (C) Sex: 54 (M), 40 (F)	Not mentioned	E: 0.5–0.9: 2; 1–3: 6; > 3: 39 C: 0.5–0.9: 2; 1–3: 8; > 3: 37	Physical therapy	E: Photon therapy was performed twice a day for 20 min at each site until discharge C: health education	ultrasound examination	(1) The maximum LH of the long, wide, and deep diameters (2) The total LH of the long, wide, and deep diameters (3) The average LH of the long, wide, and deep diameters

Wang 2024 [47]	70 E: 35 C: 35	Age (years): 69.82 ± 6.10 (E), 61.09 ± 8.76 (C) Sex: 37 (M), 33 (F)	E: 6.98 ± 1.97 C: 6.56 ± 1.91	Not mentioned	Appropriate technology of Traditional Chinese Medicine	E: Hot compress Puhuang hot compress for 20 min twice a day for 41 days C: 50% magnesium sulfate wet hot compress twice a day, 20 min each time, every 5 min to change once, a total of 41 days	ultrasound examination	(1) Curative effect (2) HbA1c (3) FPG (4) HOMA‐IR (5) LH hiperplasia (6) GCQ

### 2.4. Assessment of Risk of Bias

We used the revised version of Cochrane’s tool for assessing risk of bias [48]. The risk of bias for all studies was assessed across the following domains: (1) randomization process; (2) deviation from the intended intervention; (3) missing outcome data; (4) measurement of outcomes; and (5) selection of reported outcomes. Each domain was rated as having low, moderate, or high risk of bias. The risk of bias for each study was assessed independently based on distinct assessment criteria and outcome measures. In cases of disagreement, a third researcher (WLP) was consulted to reach consensus.

### 2.5. Data Synthesis and Analysis

Results analysis was performed using Review Manager (RevMan), Version 5.3 software. Statistical significance was determined when the 95% confidence interval (CI) did not include the null value of zero. Where possible and available, intention‐to‐treat data were prioritized. For studies reporting continuous variables as quartiles and medians, we converted these to mean and standard deviation. Standardized mean difference (SMD) and 95% CIs were reported for the continuous outcomes of HbA1c, LH diameter, FBG, and 2hPG. The odds ratio (OR) and 95% CIs were reported as curative effect criteria for binary classification outcomes [49–51]. Given the multicomponent nature of noninjection intervention measures, a prior random‐effects model is preferred [52]. Statistical heterogeneity within each meta‐analysis was assessed using the Cochran Q test and *I* ^2^ statistic. A *p* value of < 0.1 or an *I* ^2^ value of  > 50% indicated significant or considerable heterogeneity [53]. To investigate publication bias, we constructed funnel plots and calculated the Egger test using the metafunnel and metabias commands in Stata, Version 12.0 [54]. We conducted sensitivity analyses using the leave‐one‐out method and based on bias risk to examine the robustness of the results [54].

## 3. Results

**FIGURE 1:**
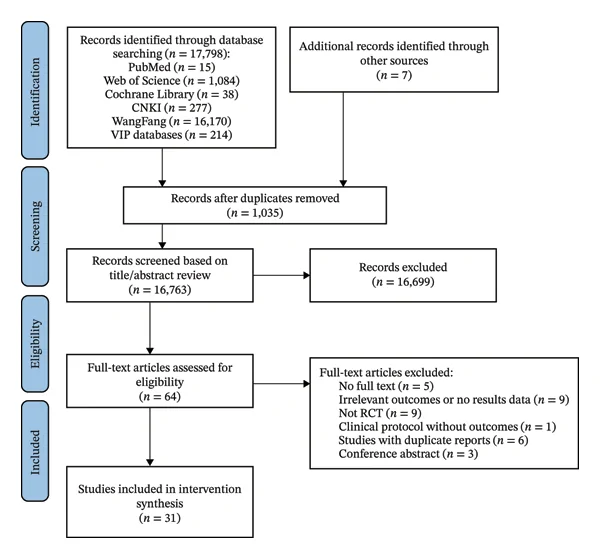
PRISMA flowcharts of the study.

The literature search retrieved 17,798 records, of which 31 studies with 3496 participants were included in the meta‐analyses [17–44, 46, 47, 55]. Figure 1 shows the search and selection of studies based on the PRISMA flowcharts. A total of 1035 studies were removed due to duplicates, and title and abstract screening resulted in the exclusion of 16,699 articles. The remaining 64 studies were retrieved for further full‐text evaluations, 33 of which were excluded because they did not meet eligibility criteria. Finally, 31 RCTs were included in this review and 27 RCTs were included in the meta‐analysis [17–29, 31–35, 37–42, 47, 55].

### 3.1. Study Characteristics

Table 1 presents the characteristics and summary findings of included studies. As the study evaluated two different noninjection technology intervention programs, we counted Ai 2013, Xiong 2017, and Ai 2020 as two studies (Ai 2013 [appropriate technology of TCM]; Ai 2013 [physical therapy]; Xiong 2017 [appropriate technology of TCM]; Xiong 2017 [physical therapy]; Ai 2020 [appropriate technology of TCM]; Ai 2020 [physical therapy]) [17, 20, 30]. Thus, a total of 2555 subjects from 34 studies were included in this study. The noninjection technology interventions included in the study included two broad categories: TCM appropriate technology and physical therapy. We counted Yu 2021 as a TCM appropriate technology because the study used hydrocolloid dressings that could not be categorized, and the principle of action of hydrocolloid dressings is similar to that of external TCM treatments in TCM appropriate technology [35]. Therefore, TCM appropriate technology included the category of TCM moxibustion (*n* = 8) and TCM external treatment (*n* = 22). Physical therapy included photon therapy (*n* = 2), infrared therapy (*n* = 1), and TDP therapy (*n* = 1). Twenty‐seven studies used the curative effect as an outcome measure and were included in a meta‐analysis. Intervention cycles ranged from 5 days to 3 months. Five studies used HbA1c as an outcome measure and included in a meta‐analysis [18, 26, 29, 42, 47]. Five studies used FBG as an outcome measure and were included in a meta‐analysis. Three studies used 2hPG as an outcome measure and were included in a meta‐analysis. Three studies categorized the LH diameter conditions into mild, moderate, and severe, which were subjected to meta‐analysis.

The overall certainty of evidence (Grading of Recommendations Assessment, Development, and Evaluation) for each outcome is summarized in Supporting Table 1.

### 3.2. Risk of Bias

**FIGURE 2:**
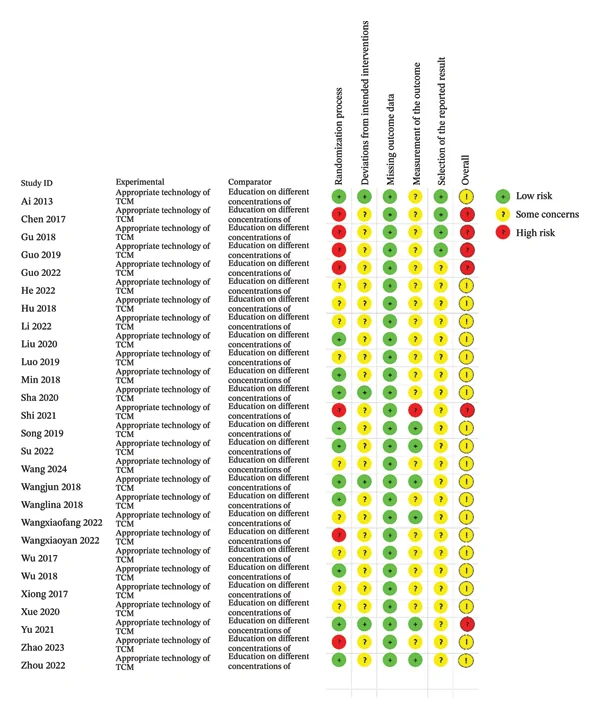
Risk of bias of included studies.

Assessments of the risk of bias are shown in Figure 2, Supporting Figure 1, and Supporting Figure 2.

### 3.3. Results of Meta‐Analysis

#### 3.3.1. Primary Outcome

##### 3.3.1.1. Curative Effect

**FIGURE 3:**
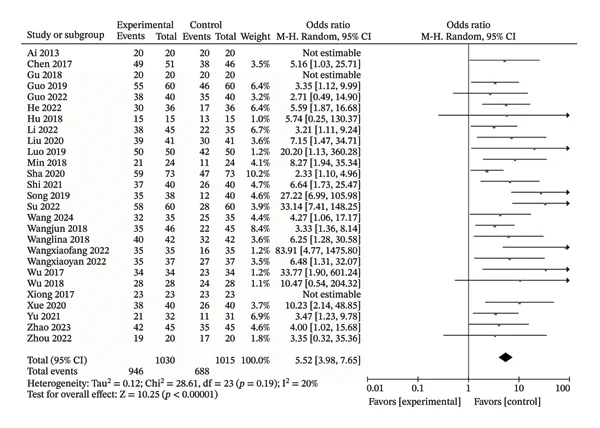
Forest plot of the effect of TCM appropriate technique intervention on the curative effect.

Twenty‐seven studies were included in the meta‐analysis to evaluate the impact of TCM on the curative effect of LH. Meta‐analysis results showed that TCM interventions improved the curative effect of LH (OR = 5.52; 95% CI = 3.98 to 7.65; *p* < 0.00001) (Figure 3). There was negligible heterogeneity (*p* = 0.19, *I* ^2^ = 20%) (Supporting Figure 3).

###### 3.3.1.1.1. Intervention Duration

Subgroup analysis showed that intervention duration of ≤ 7 days, 7–14 days, and > 14 days had a significant effect on LH treatment (Supporting Figure 4).

###### 3.3.1.1.2. Detection Method

Subgroup analysis was performed according to the detection method (palpation/visual inspection vs. ultrasound examination). Both subgroups demonstrated positive effects, with the palpation/visual inspection subgroup showing a pooled OR of 5.52 (95% CI: 3.98–7.65) with no heterogeneity (*I* ^2^ = 0%, *p* = 0.87) and the ultrasound subgroup showing a pooled OR of 10.78 (95% CI: 3.82–30.38) with moderate heterogeneity (*I* ^2^ = 63%, *p* = 0.02). Notably, significant heterogeneity was observed within the ultrasound subgroup (Supporting Figure 5). Given the inherent differences in measurement accuracy between these diagnostic approaches, these subgroup findings are presented for descriptive purposes only, and direct statistical comparisons between subgroups were not performed.

To specifically address this heterogeneity and to explicitly evaluate the methods recommended by the fitter document, we performed a sensitivity analysis restricted to the six ultrasound‐based studies, using a leave‐one‐out approach.

Sensitivity analysis confirmed the robustness of the ultrasound‐restricted findings: On the lnOR scale, after sequentially excluding each study, the pooled estimates remained stable, ranging from 1.60 to 2.00, with all 95% CIs excluding the null (lnOR = 0), indicating that no single study exerted undue influence on the overall estimate.

###### 3.3.1.1.3. Suitable Technical Categories for TCM

When we grouped the studies according to the suitable technical categories for TCM, we found that both TCM moxibustion and TCM external treatments were effective for LH (Supporting Figure 6). There was less heterogeneity across subgroups. Compared with TCM moxibustion, the effect of external TCM treatment on LH efficacy may be more significant.

###### 3.3.1.1.4. Diabetes Type

Subgroup analysis was also performed according to the diabetes type. Both subgroup analyses demonstrated highly significant overall effects (*p* < 0.00001 for both). Although the subgroup of studies with unspecified diabetes type showed no heterogeneity (*I* ^2^ = 0%), while the T2DM‐specified subgroup showed moderate heterogeneity (*I* ^2^ = 51%), the overall treatment effect remained robust and significant across both subgroups (Supporting Figure 10).

#### 3.3.2. Secondary Outcomes

##### 3.3.2.1. HbA1c

**FIGURE 4:**
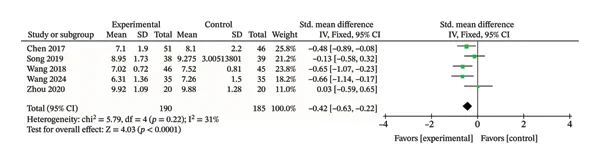
Forest plot of the effect of TCM appropriate technique intervention on HbA1c.

The meta‐analysis of the change in HbA1c included five studies. The results of the meta‐analysis showed a positive effect of the TCM intervention on HbA1c (SMD = −0.42; 95% CI = −0.63 to −0.22; *p* < 0.0001), with negligible heterogeneity (*p* = 0.22, *I* ^2^ = 31%) (Figure 4).

##### 3.3.2.2. FBG

Five studies were included in the meta‐analysis to evaluate the impact of the appropriate technology of TCM interventions on the FBG levels of LH people. Meta‐analysis results showed that appropriate technology of TCM interventions improved the FBG of LH people (SMD = −0.70; 95% CI = −1.41 to 0.01; *p* < 0.0001). There was high heterogeneity (*p* < 0.00001, I^2^ = 91%) (Supporting Figure 7). Although the funnel plot exhibited asymmetry, there was no significant publication bias based on Egger’s test (*p* = 0. 354) (Supporting Figure 8).

##### 3.3.2.3. 2hPG

Three studies were included in the meta‐analysis to evaluate the impact of the appropriate technology of TCM interventions on the 2hPG levels of LH people. Meta‐analysis results showed that appropriate technology of TCM interventions improved the FBG of LH people (SMD = −1.14; 95% CI = −2.44 to 0.16; *p* < 0.0001), with high heterogeneity (*p* < 0.00001, *I* ^2^ = 94%) (Supporting Figure 9).

## 4. Discussion

To our knowledge, this is the first meta‐analysis of RCTs investigating noninjection technique education, aiming to explore the effectiveness of noninjection technique education for LH. The analysis of the included studies showed that the noninjection technical education was mainly on appropriate Chinese medicine techniques and physical therapy, the interventions were mainly for inpatients, and the testing techniques were based on visual and tactile diagnostic methods.

This study explored the effects of appropriate TCM techniques and physical therapy on HbA1, LH severity, FBG, and 2hPG. The included studies demonstrated that TCM appropriate techniques and physical therapy promoted LH recovery, improved moderate‐to‐severe LH, reduced HbA1c levels, and improved the QoL of people with diabetes, compared with educational interventions on injection techniques, routine care, and magnesium sulfate wet heat packs. Regarding their effects on FBG and 2hPG levels, the evidence provided was inconclusive due to high heterogeneity.

Theoretically, moxibustion involves the direct or indirect application of ignited moxa or other medicinal substances to acupuncture points on the body surface. Through the heat generated by moxa combustion and the pharmacological effects of the herbs, the therapeutic effects are transmitted along the meridians. These effects include warming qi and blood, unblocking meridians, harmonizing yin and yang, reinforcing the body’s defensive qi to eliminate pathogenic factors, promoting the flow of vital energy and blood, expelling cold and dampness, and reducing swelling and dissipating nodules. The external therapy of Chinese medicine uses external application therapy and plaster therapy to dredge the meridians, activate blood circulation and remove blood stasis, dissipate cold and dampness, regulate the internal organs, and prevent and cure diseases. Therefore, the TCM appropriate technology can promote the dissipation of LH to improve the effect of LH, thus reducing the levels of HbA1c, FBG, and 2hPG, which can improve the QoL of diabetic people.

Through subgroup analysis, we found that the effect of intervention duration > 14 days was better than that of < 7 days and 7–14 days. In addition to the appropriate technical intervention of TCM, another possible reason is that through intervention, the patient avoided repeated injections at the LH site and passively rotated the injection site multiple times. The ultrasound hypothesis can be used as a basis to avoid injection at the LH site, as the LH will gradually shrink or disappear.

Ai et al. intervened in LH with physical therapy (infrared therapy and microwave therapy) combined with TCM appropriate techniques, whereas the study by Xu et al. used only physical therapy (photon therapy) as an intervention, both of which had some intervention effect. However, the data could not be analyzed together due to variable outcome indicators, while the interpretation of the intervention effect of physical therapy on LH needs to be cautious due to the combination of interventions.

The findings of this study, together with the inherent limitations of education‐based interventions (e.g., delayed therapeutic response and declining adherence over time), highlight the potential value of adjunctive therapeutic strategies for LH management. Among these, physical therapies and TCM external treatments have attracted increasing clinical interest. Modalities such as ultrasound therapy, Chinese herbal moist heat packs, and acupoint application have been reported in some observational studies and small case series to provide symptomatic relief, possibly through mechanisms involving improved local microcirculation, anti‐inflammatory effects, and promotion of adipose tissue remodeling. [53, 54] However, it must be acknowledged that the current evidence base remains weak; most available data derive from uncontrolled studies or small‐sample investigations, and high‐quality RCTs are conspicuously lacking. Key unresolved issues include the standardization of treatment protocols (e.g., optimal concentration, temperature, frequency, and duration), the selection of appropriate outcome measures, and the establishment of ultrasound‐based objective efficacy criteria. Future research should prioritize rigorously designed, adequately powered RCTs with homogeneous diagnostic standards (i.e., ultrasound‐confirmed LH) and validated assessment tools. Such studies would not only clarify the true therapeutic value of these modalities but also provide a replicable framework for clinical practice and further investigation.

Our findings should be interpreted with consideration of the diagnostic heterogeneity across the included studies. The predominance of palpation‐based diagnosis in the current literature (21/27 studies, 77.8%) reflects a methodological gap. A variation in diagnostic criteria may complicate cross‐study comparisons and influence the reliability of efficacy estimates. Nevertheless, our sensitivity analysis restricted to the 6 ultrasound‐based studies showed results generally comparable to the primary analysis, which provides some reassurance that diagnostic heterogeneity may not have substantially affected our main conclusions. These observations highlight the value of future studies adopting standardized ultrasound‐based diagnostic and outcome assessment methods to further strengthen the evidence base.

Some limitations of this study should be mentioned. First, the overall risk of bias of the included studies was mainly “high” or “some concerns,” and the results need to be interpreted with caution. Although we used a random‐effects model and conducted multiple subgroup analyses, variations existed across included studies in terms of the specific details of TCM appropriate techniques (e.g., type of manipulation, treatment intensity, acupoint selection, and treatment duration) as well as diagnostic criteria for LH. These variations may affect the robustness of the pooled effect estimates. Second, the generalizability and applicability of our findings are limited, and the included studies were mainly from China, with few studies from other regions. Third, fewer studies were included in the meta‐analysis that included HbAc1, FBG, and 2hPG as outcomes, so the strength of evidence for the results is limited. Additionally, some studies may not have been included due to language restrictions or unavailability of full texts, which may introduce reporting bias. Fourth, palpable‐only lesions without ultrasound confirmation may introduce measurement bias. Although our sensitivity analysis restricted to the six ultrasound‐based studies yielded results consistent with the primary analysis, the small number of such studies (*n* = 6) limits the statistical power of this analysis.

## 5. Conclusion

Existing evidence suggests that TCM appropriate techniques and physical therapy in noninjectable technology education may be effective for people with LH. However, as they are not at the same frequency of intervention, the durability and economy of the intervention are not clear for the time being, and these factors need to be fully considered in the future, and it is hoped that large‐scale, multicenter, low‐risk‐of‐bias RCTs can be conducted to accurately assess efficacy and feasibility.

## Author Contributions

Liqing Jiang, Honglian Fang, Xumei Tao, and Liping Wang were involved in the design and concept of this study. Liqing Jiang and Honglian Fang conducted the systematic literature search and data extraction and wrote this article. Liqing Jiang and Honglian Fang conducted the analyses. Xumei Tao was involved in the systematic literature search, and Liping Wang was involved in data extraction. Liqing Jiang and Liping Wang contributed to the drafting of the final article.

## Funding

This study was supported by the Science and Technology Development Fund of Nanjing Medical University Commission, No. NMU20230181.

## Conflicts of Interest

The authors declare no conflicts of interest.

## Supporting Information

Additional supporting information can be found online in the Supporting Information section.

## Supporting information


**Supporting Information** Supporting Table 1. The overall certainty of each outcome. Supporting Figure 1: Risk of bias of included studies about the outcome indicator HbA1c. Supporting Figure 2: Risk of bias of included studies about the outcome indicator LH diameter. Supporting Figure 3: Sensitivity analysis for curative effect by excluding studies by turns. Supporting Figure 4: TCM appropriate techniques—subgroup analysis based on intervention duration. Supporting Figure 5: Subgroup analysis based on the detection method and sensitivity analysis restricted to ultrasound‐based diagnostic studies. Supporting Figure 6: TCM appropriate techniques—subgroup analysis based on suitable technical categories. Supporting Figure 7: Forest plot of the effect of TCM appropriate technique intervention on FBG. Supporting Figure 8: Egger’s test on FBG. Supporting Figure 9: Forest plot of the effect of TCM appropriate technique intervention on 2hPG. Supporting Figure 10: TCM appropriate techniques—subgroup analysis based on diabetes classification. Supporting Information 1: PRISMA checklist. Supporting Information 2: Search strategies.

## Data Availability

The authors confirm that the data supporting the findings of this study are available within the article and Supporting information.
