# Supplementary material for: Efficacy of Appropriate Techniques of Traditional Chinese Medicine and Physical Therapy in Patients With Insulin‐Associated Lipohypertrophy: A Systematic Review and Meta‐Analysis
Source: Int J Endocrinol. 2026 Aug 27;2026:5589949. doi: 10.1155/ije/5589949 (PMC13520793; doi:10.1155/ije/5589949)
Supplement: Supplementary file 1 [file IJE-2026-5589949-s001.zip › Supplemental figure 1 Risk of bias of included studies about the outcome indicator HbA1c..pdf]

Study ID	Experimental	Comparator	Randomization process	Deviations from intended interventions	Missing outcome data	Measurement of the outcome	Selection of the reported result	Overall
Chen 2017	Appropriate technology of	Education on different	?	?	+	?	+	?
Song 2019	Appropriate technology of	Education on different	+	?	+	+	?	!
Su 2022	Appropriate technology of	Education on different	+	?	+	+	?	!
Wang 2024	Appropriate technology of	Education on different	?	?	+	?	?	!
Wangjun 2018	Appropriate technology of	Education on different	+	+	+	+	?	!
Zhou 2020	Appropriate technology of	Education on different	?	?	+	?	?	!

+ Low risk

? Some concerns

? High risk
