# Supplementary material for: Efficacy of Appropriate Techniques of Traditional Chinese Medicine and Physical Therapy in Patients With Insulin‐Associated Lipohypertrophy: A Systematic Review and Meta‐Analysis
Source: Int J Endocrinol. 2026 Aug 27;2026:5589949. doi: 10.1155/ije/5589949 (PMC13520793; doi:10.1155/ije/5589949)

Study or Subgroup	Experimental Events	Experimental Total	Control Events	Control Total	Weight	Odds Ratio M-H, Fixed, 95% CI
**1.2.1 T2DM**						
Chen 2017	49	51	38	46	3.1%	5.16 [1.03, 25.71]
Guo 2019	55	60	46	60	7.7%	3.35 [1.12, 9.99]
Guo 2022	38	40	35	40	3.5%	2.71 [0.49, 14.90]
He 2022	30	36	17	36	5.7%	5.59 [1.87, 16.68]
Li 2022	38	45	22	35	7.7%	3.21 [1.11, 9.24]
Luo 2019	50	50	42	50	0.8%	20.20 [1.13, 360.28]
Sha 2020	59	73	47	73	18.0%	2.33 [1.10, 4.96]
Song 2019	35	38	12	49	1.7%	35.97 [9.35, 138.33]
Su 2022	58	60	28	60	1.9%	33.14 [7.41, 148.25]
Wang 2024	32	35	25	35	4.3%	4.27 [1.06, 17.17]
Wangjun 2018	35	46	22	45	10.6%	3.33 [1.36, 8.14]
Wu 2018	34	34	23	34	0.7%	33.77 [1.90, 601.24]
Yu 2021	21	32	11	31	7.7%	3.47 [1.23, 9.78]
**Subtotal (95% CI)**		**600**		**594**	**73.3%**	**5.33 [3.89, 7.31]**
Total events	534		368			
Heterogeneity: Chi² = 24.47, df = 12 (P = 0.02); I² = 51%						
Test for overall effect: Z = 10.39 (P < 0.00001)						
**1.2.2 DM**						
Ai 2013	20	20	20	20		Not estimable
Gu 2018	20	20	20	20		Not estimable
Hu 2018	15	15	13	15	0.8%	5.74 [0.25, 130.37]
Liu 2020	39	41	30	41	2.9%	7.15 [1.47, 34.71]
Min 2018	21	24	11	24	2.8%	8.27 [1.94, 35.34]
Shi 2021	37	40	26	40	3.9%	6.64 [1.73, 25.47]
Wanglina 2018	40	42	32	42	3.0%	6.25 [1.28, 30.58]
Wangxiaofang 2022	35	35	16	35	0.5%	83.91 [4.77, 1475.80]
Wangxiaoyan 2022	35	37	27	37	2.9%	6.48 [1.31, 32.07]
Wu 2017	28	28	24	28	0.8%	10.47 [0.54, 204.32]
Xiong 2017	23	23	23	23		Not estimable
Xue 2020	38	40	26	40	2.6%	10.23 [2.14, 48.85]
Zhao 2023	42	45	35	45	4.7%	4.00 [1.02, 15.68]
Zhou 2022	19	20	17	20	1.7%	3.35 [0.32, 35.36]
**Subtotal (95% CI)**		**430**		**430**	**26.7%**	**7.90 [4.76, 13.13]**
Total events	412		320			
Heterogeneity: Chi² = 4.48, df = 10 (P = 0.92); I² = 0%						
Test for overall effect: Z = 7.98 (P < 0.00001)						
**Total (95% CI)**		**1030**		**1024**	**100.0%**	**6.02 [4.60, 7.86]**
Total events	946		688			
Heterogeneity: Chi² = 30.90, df = 23 (P = 0.13); I² = 26%						
Test for overall effect: Z = 13.15 (P < 0.00001)						
Test for subgroup differences: Chi² = 1.66, df = 1 (P = 0.20), I² = 39.9%						

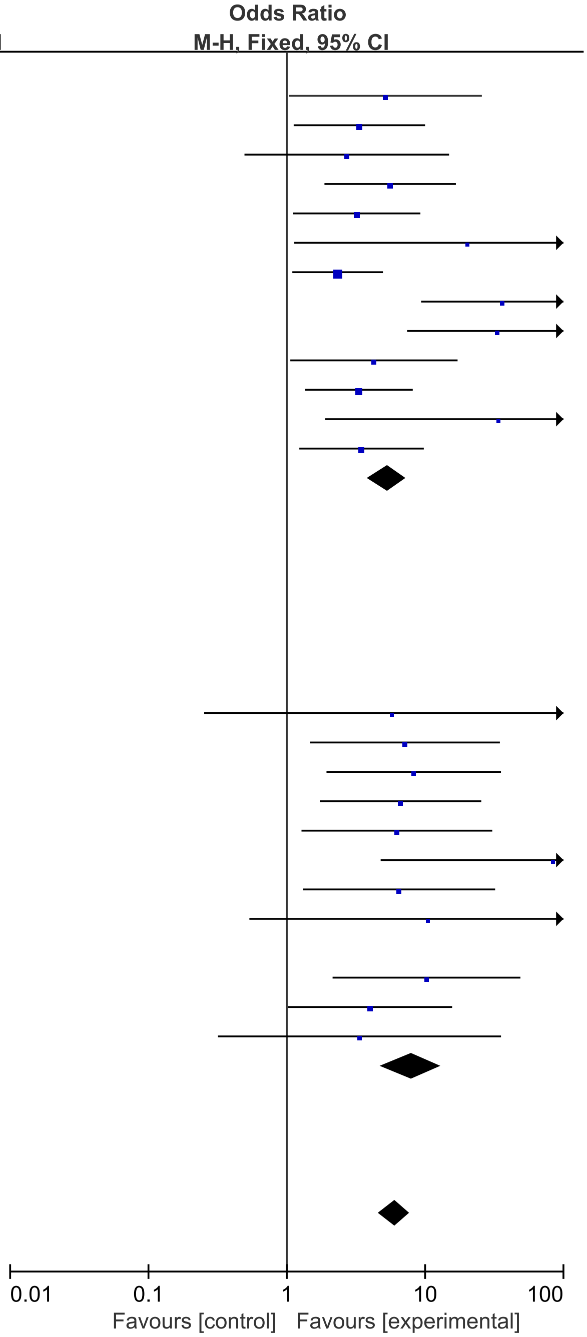

Supplement: Supplementary file 1 [file IJE-2026-5589949-s001.zip › Supplemental figure 10 TCM appropriate techniques-subgroup analysis based on diabetes classificat.pdf]
