# Supplementary material for: Efficacy of Appropriate Techniques of Traditional Chinese Medicine and Physical Therapy in Patients With Insulin‐Associated Lipohypertrophy: A Systematic Review and Meta‐Analysis
Source: Int J Endocrinol. 2026 Aug 27;2026:5589949. doi: 10.1155/ije/5589949 (PMC13520793; doi:10.1155/ije/5589949)
Supplement: Supplementary file 1 [file IJE-2026-5589949-s001.zip › Supplemental figure 2 Risk of bias of included studies about the outcome indicator LH diameter..pdf]

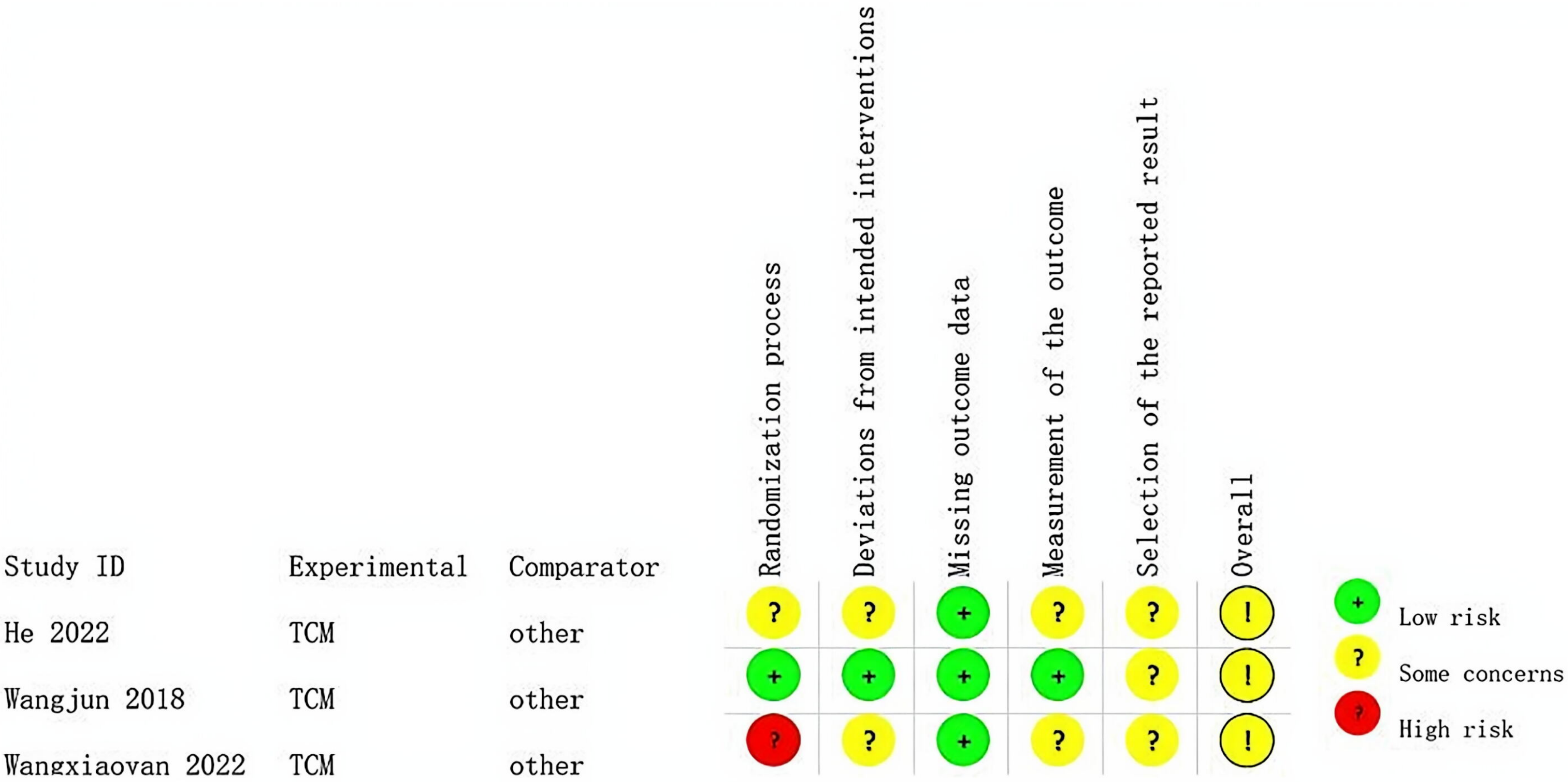

Randomization process
Deviations from intended interventions
Missing outcome data
Measurement of the outcome
Selection of the reported result
Overall
Study ID
Experimental
Comparator
He 2022
TCM
other
Wangjun 2018
TCM
other
Wangxiaovan 2022
TCM
other
Low risk
Some concerns
High risk
