# Supplementary material for: Efficacy of Appropriate Techniques of Traditional Chinese Medicine and Physical Therapy in Patients With Insulin‐Associated Lipohypertrophy: A Systematic Review and Meta‐Analysis
Source: Int J Endocrinol. 2026 Aug 27;2026:5589949. doi: 10.1155/ije/5589949 (PMC13520793; doi:10.1155/ije/5589949)
Supplement: Supplementary file 1 [file IJE-2026-5589949-s001.zip › Supplemental figure 3 Sensitivity analysis for curative effect by excluding studies by turns..pdf]

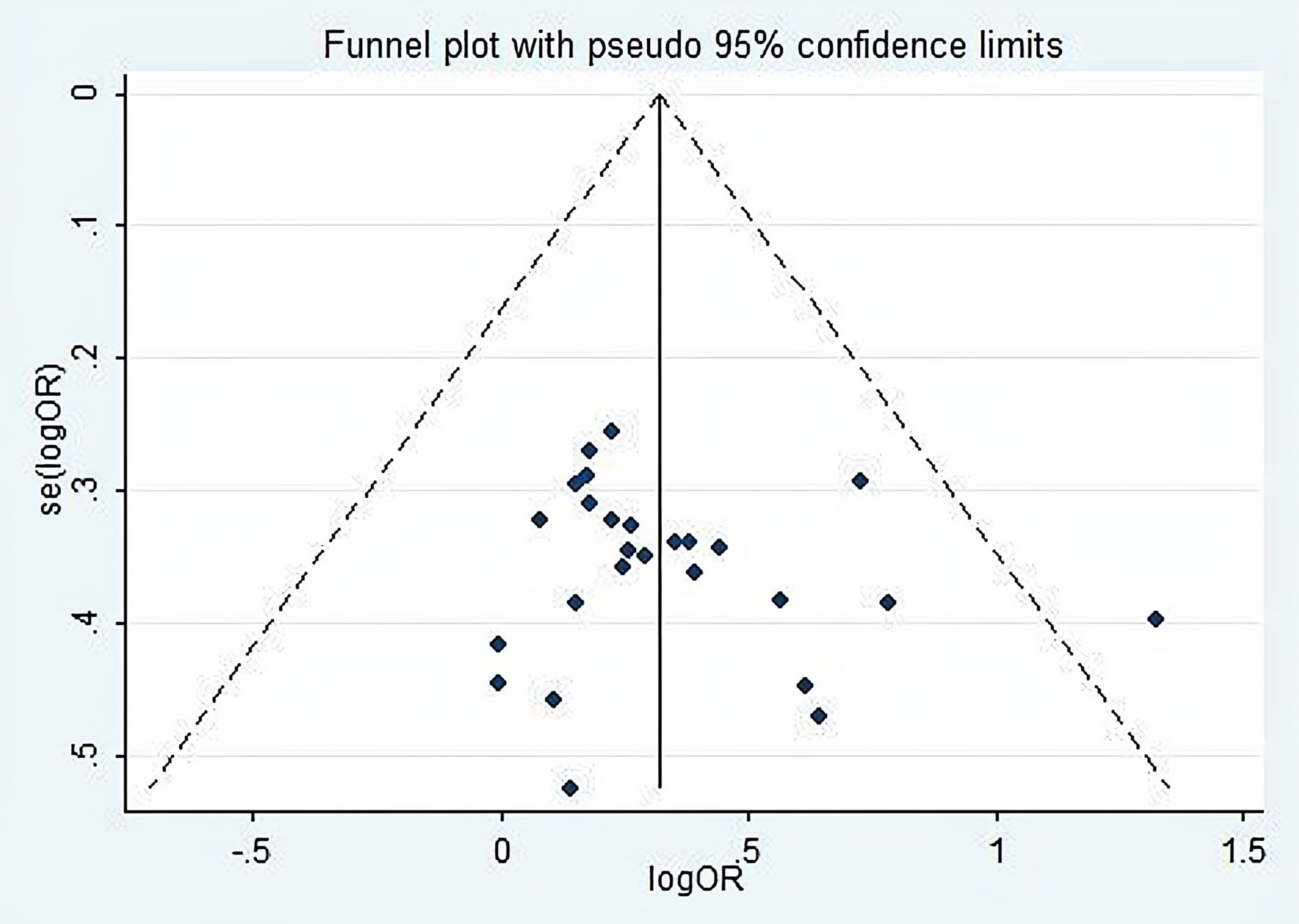
Funnel plot with pseudo 95% confidence limits
se(logOR)
0
.1
.2
.3
.4
.5
-.5
0
.5
1
1.5
logOR

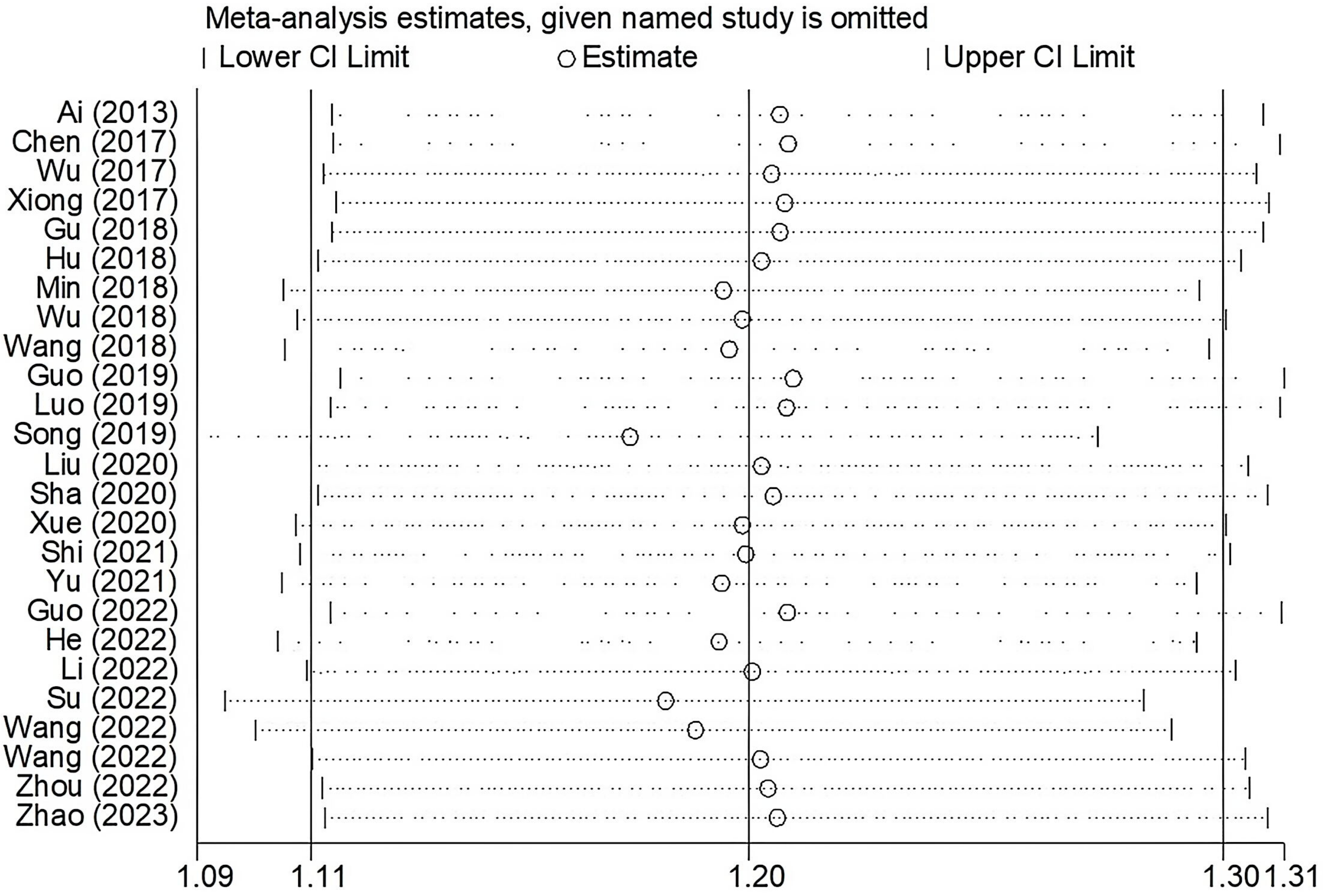
Meta-analysis estimates, given named study is omitted
Lower CI Limit
Estimate
Upper CI Limit
Ai (2013)
Chen (2017)
Wu (2017)
Xiong (2017)
Gu (2018)
Hu (2018)
Min (2018)
Wu (2018)
Wang (2018)
Guo (2019)
Luo (2019)
Song (2019)
Liu (2020)
Sha (2020)
Xue (2020)
Shi (2021)
Yu (2021)
Guo (2022)
He (2022)
Li (2022)
Su (2022)
Wang (2022)
Wang (2022)
Zhou (2022)
Zhao (2023)
1.09
1.11
1.20
1.30
1.31
