# Supplementary material for: Efficacy of Appropriate Techniques of Traditional Chinese Medicine and Physical Therapy in Patients With Insulin‐Associated Lipohypertrophy: A Systematic Review and Meta‐Analysis
Source: Int J Endocrinol. 2026 Aug 27;2026:5589949. doi: 10.1155/ije/5589949 (PMC13520793; doi:10.1155/ije/5589949)

Study or Subgroup	Experimental Events	Experimental Total	Control Events	Control Total	Weight	Odds Ratio M-H, Random, 95% CI
**1.2.1 ≤7d**						
Guo 2022	55	60	46	60	6.4%	3.35 [1.12, 9.99]
Luo 2019	50	50	42	50	1.2%	20.20 [1.13, 360.28]
Min 2018	21	24	11	24	4.1%	8.27 [1.94, 35.34]
Wanglina 2018	40	42	32	42	3.6%	6.25 [1.28, 30.58]
Wangxiaoyan 2022	35	37	27	37	3.5%	6.48 [1.31, 32.07]
**Subtotal (95% CI)**		**213**		**213**	**18.8%**	**5.63 [2.87, 11.01]**
Total events	201		158			
Heterogeneity: Tau² = 0.00; Chi² = 1.97, df = 4 (P = 0.74); I² = 0%						
Test for overall effect: Z = 5.04 (P < 0.00001)						
**1.2.2 7-14d**						
Chen 2017	49	51	38	46	3.5%	5.16 [1.03, 25.71]
Gu 2018	20	20	20	20		Not estimable
Li 2022	38	45	22	35	6.7%	3.21 [1.11, 9.24]
Liu 2020	39	41	30	41	3.6%	7.15 [1.47, 34.71]
Su 2022	58	60	28	60	3.9%	33.14 [7.41, 148.25]
Wangjun 2018	35	46	22	45	8.4%	3.33 [1.36, 8.14]
Wangxiaoyan 2022	35	35	16	35	1.2%	83.91 [4.77, 1475.80]
Wu 2018	34	34	23	34	1.2%	33.77 [1.90, 601.24]
Xiong 2017	23	23	23	23		Not estimable
Xue 2020	38	40	26	40	3.7%	10.23 [2.14, 48.85]
Yu 2021	21	32	11	31	6.9%	3.47 [1.23, 9.78]
**Subtotal (95% CI)**		**427**		**410**	**39.1%**	**7.03 [3.61, 13.72]**
Total events	390		259			
Heterogeneity: Tau² = 0.46; Chi² = 15.36, df = 8 (P = 0.05); I² = 48%						
Test for overall effect: Z = 5.72 (P < 0.00001)						
**1.2.3 ＞14d**						
Ai 2013	20	20	20	20		Not estimable
Guo 2019	38	40	35	40	3.2%	2.71 [0.49, 14.90]
He 2022	30	36	17	36	6.4%	5.59 [1.87, 16.68]
Hu 2018	15	15	13	15	1.0%	5.74 [0.25, 130.37]
Sha 2020	59	73	47	73	10.2%	2.33 [1.10, 4.96]
Shi 2021	37	40	26	40	4.7%	6.64 [1.73, 25.47]
Song 2019	35	38	12	40	4.6%	27.22 [6.99, 105.98]
Wang 2024	32	35	25	35	4.4%	4.27 [1.06, 17.17]
Wu 2017	28	28	24	28	1.1%	10.47 [0.54, 204.32]
Zhao 2023	42	45	35	45	4.6%	4.00 [1.02, 15.68]
Zhou 2022	19	20	17	20	1.8%	3.35 [0.32, 35.36]
**Subtotal (95% CI)**		**390**		**392**	**42.0%**	**4.82 [2.93, 7.94]**
Total events	355		271			
Heterogeneity: Tau² = 0.11; Chi² = 10.96, df = 9 (P = 0.28); I² = 18%						
Test for overall effect: Z = 6.19 (P < 0.00001)						
**Total (95% CI)**		**1030**		**1015**	**100.0%**	**5.52 [3.98, 7.65]**
Total events	946		688			
Heterogeneity: Tau² = 0.12; Chi² = 28.61, df = 23 (P = 0.19); I² = 20%						
Test for overall effect: Z = 10.25 (P < 0.00001)						
Test for subgroup differences: Chi² = 0.79, df = 2 (P = 0.67), I² = 0%						

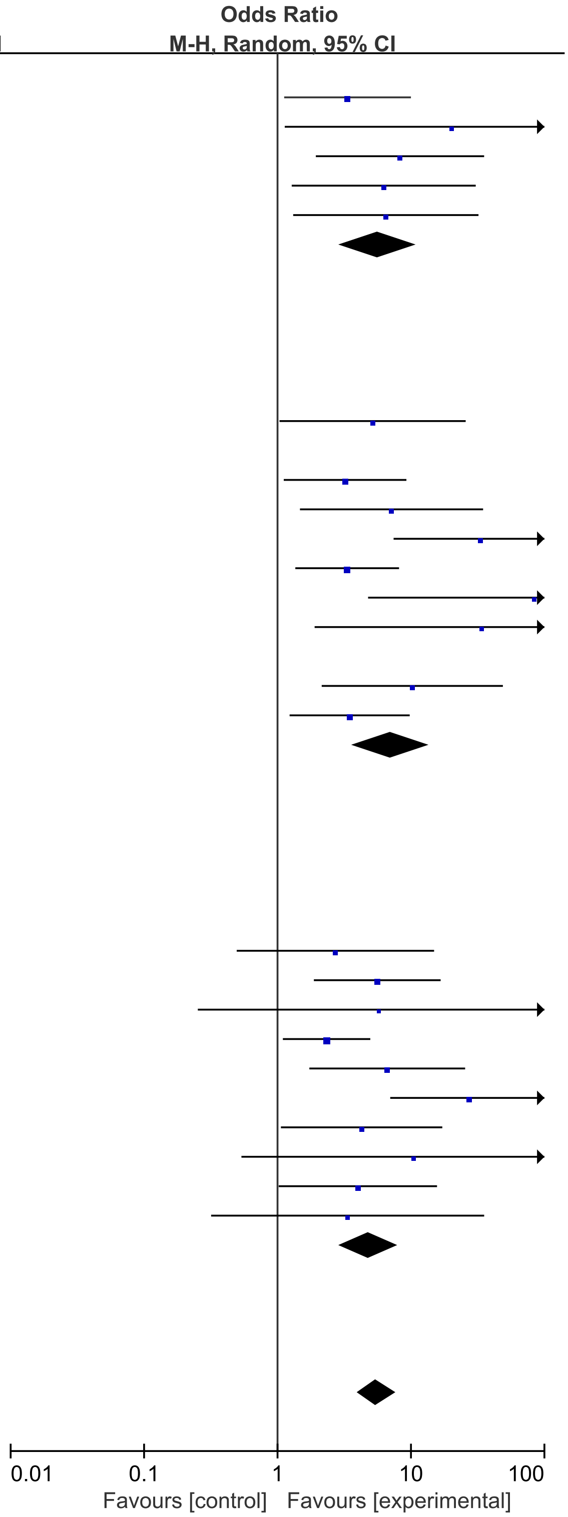

Supplement: Supplementary file 1 [file IJE-2026-5589949-s001.zip › Supplemental figure 4 TCM appropriate techniques-subgroup analysis based on intervention duration.pdf]
