# Supplementary material for: Efficacy of Appropriate Techniques of Traditional Chinese Medicine and Physical Therapy in Patients With Insulin‐Associated Lipohypertrophy: A Systematic Review and Meta‐Analysis
Source: Int J Endocrinol. 2026 Aug 27;2026:5589949. doi: 10.1155/ije/5589949 (PMC13520793; doi:10.1155/ije/5589949)
Supplement: Supplementary file 1 [file IJE-2026-5589949-s001.zip › Supplemental figure 5 Subgroup analysis based on detection method and sensitivity analysis restri.pdf]

Study or Subgroup	Experimental Events	Experimental Total	Control Events	Control Total	Weight	Odds Ratio M-H, Random, 95% CI
**1.3.1 Ultrasound examination**						
Song 2019	35	38	12	40	4.6%	27.22 [6.99, 105.98]
Su 2022	58	60	28	60	3.9%	33.14 [7.41, 148.25]
Wang 2024	32	35	25	35	4.4%	4.27 [1.06, 17.17]
Wangxiaofang 2022	35	35	16	35	1.2%	83.91 [4.77, 1475.80]
Yu 2021	21	32	11	31	6.9%	3.47 [1.23, 9.78]
Zhou 2022	19	20	17	20	1.8%	3.35 [0.32, 35.36]
**Subtotal (95% CI)**		**220**		**221**	**22.9%**	**10.82 [3.76, 31.17]**
Total events	200		109			
Heterogeneity: Tau² = 1.02; Chi² = 13.35, df = 5 (P = 0.02); I² = 63%						
Test for overall effect: Z = 4.41 (P < 0.0001)						
**1.3.2 Visible and/or palpable LH determined by healthcare**						
Ai 2013	20	20	20	20		Not estimable
Chen 2017	49	51	38	46	3.5%	5.16 [1.03, 25.71]
Gu 2018	20	20	20	20		Not estimable
Guo 2019	38	40	35	40	3.2%	2.71 [0.49, 14.90]
Guo 2022	55	60	46	60	6.4%	3.35 [1.12, 9.99]
He 2022	30	36	17	36	6.4%	5.59 [1.87, 16.68]
Hu 2018	15	15	13	15	1.0%	5.74 [0.25, 130.37]
Li 2022	38	45	22	35	6.7%	3.21 [1.11, 9.24]
Liu 2020	39	41	30	41	3.6%	7.15 [1.47, 34.71]
Luo 2019	50	50	42	50	1.2%	20.20 [1.13, 360.28]
Min 2018	21	24	11	24	4.1%	8.27 [1.94, 35.34]
Sha 2020	59	73	47	73	10.2%	2.33 [1.10, 4.96]
Shi 2021	37	40	26	40	4.7%	6.64 [1.73, 25.47]
Wangjun 2018	35	46	22	45	8.4%	3.33 [1.36, 8.14]
Wanglina 2018	40	42	32	42	3.6%	6.25 [1.28, 30.58]
Wangxiaoyan 2022	35	37	27	37	3.5%	6.48 [1.31, 32.07]
Wu 2017	28	28	24	28	1.1%	10.47 [0.54, 204.32]
Wu 2018	34	34	23	34	1.2%	33.77 [1.90, 601.24]
Xiong 2017	23	23	23	23		Not estimable
Xue 2020	38	40	26	40	3.7%	10.23 [2.14, 48.85]
Zhao 2023	42	45	35	45	4.6%	4.00 [1.02, 15.68]
**Subtotal (95% CI)**		**810**		**794**	**77.1%**	**4.39 [3.20, 6.03]**
Total events	746		579			
Heterogeneity: Tau² = 0.00; Chi² = 10.80, df = 17 (P = 0.87); I² = 0%						
Test for overall effect: Z = 9.13 (P < 0.00001)						
**Total (95% CI)**		**1030**		**1015**	**100.0%**	**5.52 [3.98, 7.65]**
Total events	946		688			
Heterogeneity: Tau² = 0.12; Chi² = 28.61, df = 23 (P = 0.19); I² = 20%						
Test for overall effect: Z = 10.25 (P < 0.00001)						
Test for subgroup differences: Chi² = 2.56, df = 1 (P = 0.11), I² = 61.0%						

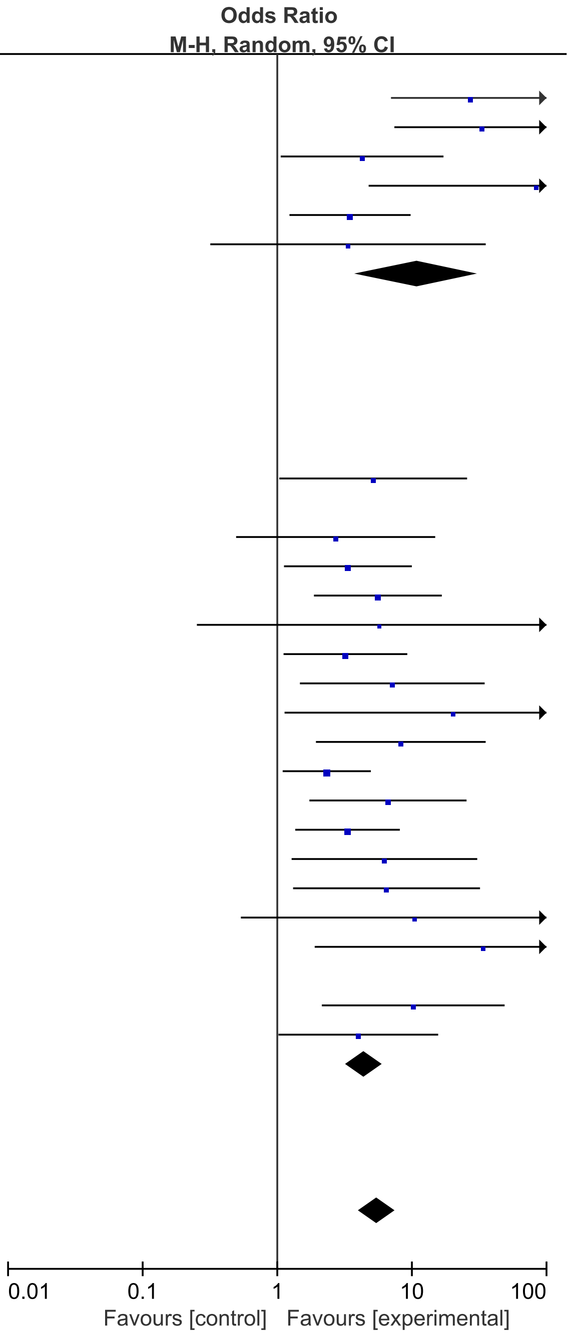

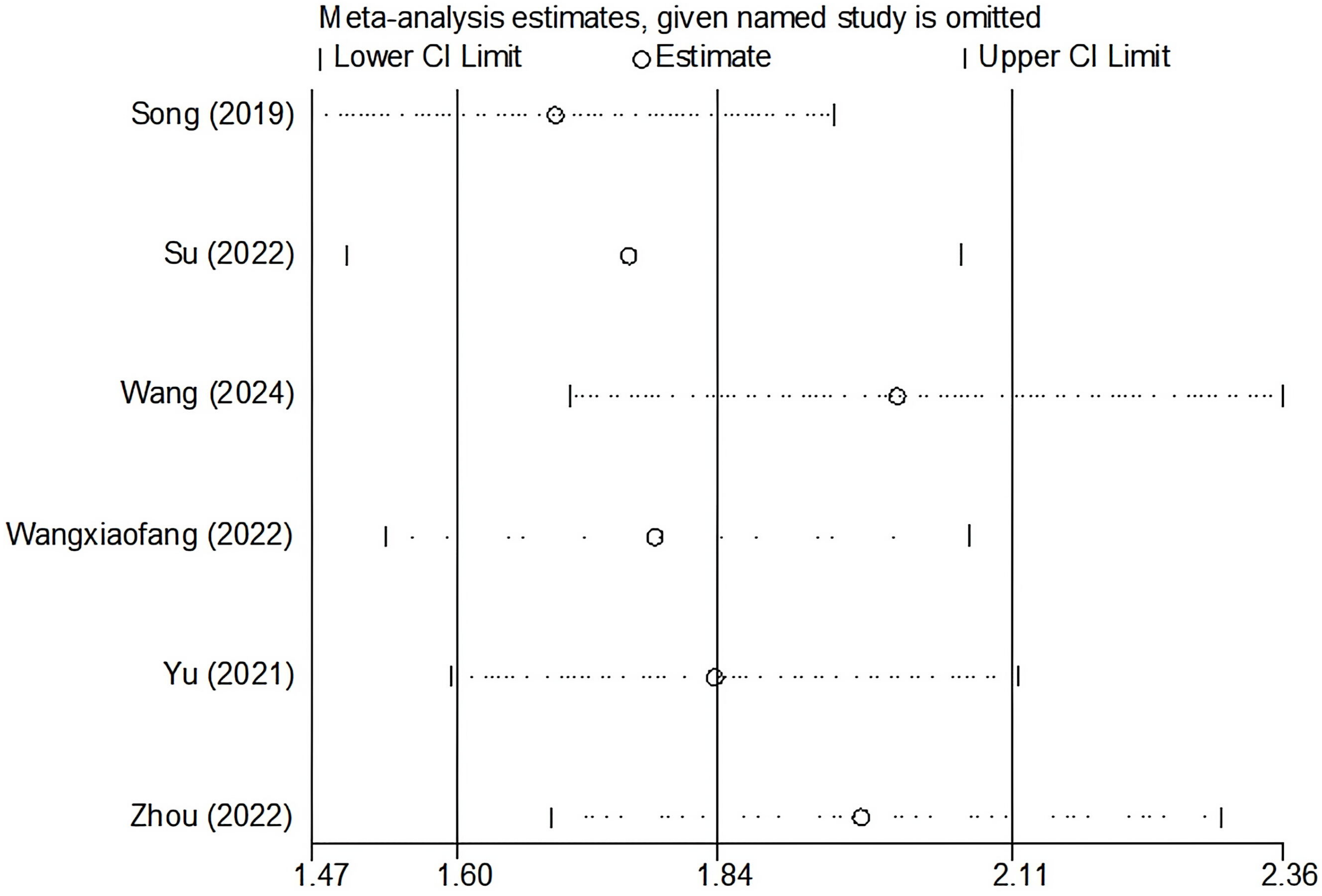

Meta-analysis estimates, given named study is omitted
Lower CI Limit
Estimate
Upper CI Limit
Song (2019)
Su (2022)
Wang (2024)
Wangxiaofang (2022)
Yu (2021)
Zhou (2022)
1.47
1.60
1.84
2.11
2.36
