# Supplementary material for: Efficacy of Appropriate Techniques of Traditional Chinese Medicine and Physical Therapy in Patients With Insulin‐Associated Lipohypertrophy: A Systematic Review and Meta‐Analysis
Source: Int J Endocrinol. 2026 Aug 27;2026:5589949. doi: 10.1155/ije/5589949 (PMC13520793; doi:10.1155/ije/5589949)

	Experimental			Control				Std. Mean Difference
Study or Subgroup	Mean	SD	Total	Mean	SD	Total	Weight	IV, Random, 95% CI
Song 2019	8.95	1.73221297	38	9.275	2.88518517	49	20.6%	-0.13 [-0.56, 0.29]
Su 2022	6.37	1.74	60	7.01	1.19	60	21.0%	-0.43 [-0.79, -0.06]
Wang 2018	7.26	1.54	46	7.95	1.63	45	20.6%	-0.43 [-0.85, -0.02]
Xu 2023	6.11	0.78	35	8.67	1.14	35	18.8%	-2.59 [-3.24, -1.95]
Zhou 2022	9.81	1.28	20	9.86	1.38	20	19.0%	-0.04 [-0.66, 0.58]
**Total (95% CI)**			**199**			**209**	**100.0%**	**-0.70 [-1.41, 0.01]**

Heterogeneity: Tau² = 0.59; Chi² = 45.72, df = 4 (P < 0.00001); I² = 91%
Test for overall effect: Z = 1.93 (P = 0.05)

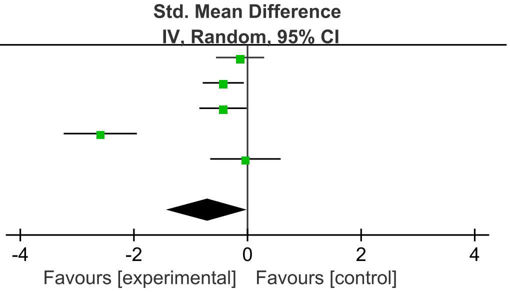

Supplement: Supplementary file 1 [file IJE-2026-5589949-s001.zip › Supplemental figure 7 Forest plot of the effect of TCM appropriate techniques intervention on FBG.pdf]
