# Supplementary material for: Efficacy of Appropriate Techniques of Traditional Chinese Medicine and Physical Therapy in Patients With Insulin‐Associated Lipohypertrophy: A Systematic Review and Meta‐Analysis
Source: Int J Endocrinol. 2026 Aug 27;2026:5589949. doi: 10.1155/ije/5589949 (PMC13520793; doi:10.1155/ije/5589949)
Supplement: Supplementary file 1 [file IJE-2026-5589949-s001.zip › Supplemental figure 8 Eggers test on FBG..pdf]

Egger's test

Std_Eff	Coef.	Std. Err.	t	P>|t|	[95% Conf. Interval]	
slope	1.177224	1.608579	0.73	0.517	-3.941991	6.296439
bias	-7.553559	6.906205	-1.09	0.354	-29.53219	14.42507
