# Supplementary material for: Efficacy of Appropriate Techniques of Traditional Chinese Medicine and Physical Therapy in Patients With Insulin‐Associated Lipohypertrophy: A Systematic Review and Meta‐Analysis
Source: Int J Endocrinol. 2026 Aug 27;2026:5589949. doi: 10.1155/ije/5589949 (PMC13520793; doi:10.1155/ije/5589949)
Supplement: Supplementary file 1 [file IJE-2026-5589949-s001.zip › Supplemental figure 9 Forest plot of the effect of TCM appropriate techniques intervention on 2hP.pdf]

	Experimental			Control				Std. Mean Difference	Std. Mean Difference
Study or Subgroup	Mean	SD	Total	Mean	SD	Total	Weight	IV, Random, 95% CI	IV, Random, 95% CI
Su 2022	7.21	1.83	60	8.12	1.09	60	34.5%	-0.60 [-0.97, -0.23]	
Xu 2023	6.98	1	35	9.76	1.08	35	32.6%	-2.64 [-3.29, -1.99]	
Zhou 2022	12.38	1.61	20	12.72	1.53	20	32.8%	-0.21 [-0.83, 0.41]	
**Total (95% CI)**			**115**			**115**	**100.0%**	**-1.14 [-2.44, 0.16]**	

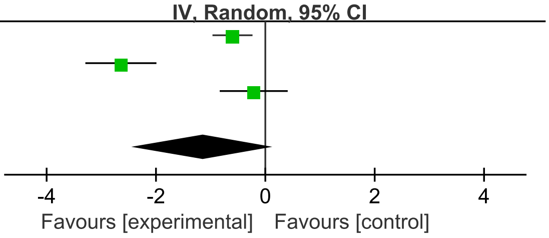

Heterogeneity: Tau² = 1.23; Chi² = 34.86, df = 2 (P < 0.00001); I² = 94%
Test for overall effect: Z = 1.72 (P = 0.09)
