# Supplementary material for: Efficacy of Appropriate Techniques of Traditional Chinese Medicine and Physical Therapy in Patients With Insulin‐Associated Lipohypertrophy: A Systematic Review and Meta‐Analysis
Source: Int J Endocrinol. 2026 Aug 27;2026:5589949. doi: 10.1155/ije/5589949 (PMC13520793; doi:10.1155/ije/5589949)
Supplement: Supplementary file 1 [file IJE-2026-5589949-s001.zip › Supplemental figures_1.docx]

Supplemental figure 1 Risk of bias of included studies about the outcome indicator HbA1c.

Supplemental figure 2 Risk of bias of included studies about the outcome indicator LH diameter.

Supplemental figure 3 Sensitivity analysis for curative effect by excluding studies by turns.

Supplemental figure 4 TCM appropriate techniques-subgroup analysis based on intervention duration.

Supplemental figure 5 TCM appropriate techniques-subgroup analysis based on detection method.

Supplemental figure 6 TCM appropriate techniques-subgroup analysis based on suitable technical categories.

Supplemental figure 7 Forest plot of the effect of TCM appropriate techniques intervention on FBG.

Supplemental figure 8 Egger’s test on FBG.

Supplemental figure 9 Forest plot of the effect of TCM appropriate techniques intervention on 2hPG.
