# Supplementary material for: Efficacy of Appropriate Techniques of Traditional Chinese Medicine and Physical Therapy in Patients With Insulin‐Associated Lipohypertrophy: A Systematic Review and Meta‐Analysis
Source: Int J Endocrinol. 2026 Aug 27;2026:5589949. doi: 10.1155/ije/5589949 (PMC13520793; doi:10.1155/ije/5589949)
Supplement: Supplementary file 1 [file IJE-2026-5589949-s001.zip › Supplemental material 2 Search strategies.docx]

PubMed:

#1 "Amyloidosis" [Mesh]
#2 "Amyloidoses" [Title/Abstract]

#3 "lipohypertrophy"[Title/Abstract]

#4 "insulin-derived amyloidosis"[Title/Abstract]

#5 "lipos"[Title/Abstract]

#6 "subcutaneous induration"[Title/Abstract]

#7 "subcutaneous nodules"[Title/Abstract]

#8 "insulin lipohypertrophy"[Title/Abstract]

#9 "endermic induration"[Title/Abstract]

#10 #1 OR #2 OR #3 OR #4 OR #5 OR #6 OR #7 OR #8 OR #9

#11 "Diabetes Mellitus" [Mesh]

#12 "Diabetes Mellitus, Type 2" [Mesh]
#13 "Diabetes Mellitus, Noninsulin-Dependent" [Title/Abstract]

#14 "Diabetes Mellitus, Ketosis-Resistant"[Title/Abstract]

#15 "Diabetes Mellitus, Ketosis Resistant"[Title/Abstract]

#16 "Ketosis-Resistant Diabetes Mellitus"[Title/Abstract]

#17 "Diabetes Mellitus, Non Insulin Dependent"[Title/Abstract]

#18 "Diabetes Mellitus, Non-Insulin-Dependent"[Title/Abstract]

#19 "Non-Insulin-Dependent Diabetes Mellitus"[Title/Abstract]

#20 "Diabetes Mellitus, Stable"[Title/Abstract]

#21 "Stable Diabetes Mellitus" [Title/Abstract]

#22 "Diabetes Mellitus, Type II"[Title/Abstract]

#23 "NIDDM"[Title/Abstract]

#24 "Diabetes Mellitus, Noninsulin Dependent"[Title/Abstract]

#25 "Diabetes Mellitus, Maturity-Onset"[Title/Abstract]

#26 "Diabetes Mellitus, Maturity Onset"[Title/Abstract]

#27 "Maturity-Onset Diabetes Mellitus"[Title/Abstract]

#28 "Maturity Onset Diabetes Mellitus"[Title/Abstract]

#29 "MODY"[Title/Abstract]

#30 "Diabetes Mellitus, Slow-Onset"[Title/Abstract]

#31 "Diabetes Mellitus, Slow Onset"[Title/Abstract]

#32 "Slow-Onset Diabetes Mellitus"[Title/Abstract]

#33 "Type 2 Diabetes Mellitus"[Title/Abstract]

#34 "Noninsulin-Dependent Diabetes Mellitus"[Title/Abstract]

#35 "Noninsulin Dependent Diabetes Mellitus"[Title/Abstract]

#36 "Maturity-Onset Diabetes"[Title/Abstract]

#37 "Diabetes, Maturity-Onset"[Title/Abstract]

#38 "Maturity Onset Diabetes"[Title/Abstract]

#39 "Type 2 Diabetes"[Title/Abstract]

#40 "Diabetes, Type 2"[Title/Abstract]

#41 "Diabetes Mellitus, Adult-Onset"[Title/Abstract]

#42 "Adult-Onset Diabetes Mellitus"[Title/Abstract]

#43 "Diabetes Mellitus, Adult Onset"[Title/Abstract]

#45 #12 OR #13 OR #14 OR #15 OR #16 OR #17 OR #18 OR #19 OR #20 OR #21 OR #22 OR #23 OR #24 OR #25 OR #26 OR #27 OR #28 OR #29 OR #30 OR #31 OR #32 OR #33 OR #34 OR #35 OR #36 OR #37 OR #38 OR #39 OR #40 OR #41 OR #42 OR #43

#46 " Diabetes Mellitus, Type 1" [Mesh]

#47 "Diabetes Mellitus, Insulin-Dependent"[Title/Abstract]

#48 "Diabetes Mellitus, Insulin Dependent"[Title/Abstract]

#49 "Insulin-Dependent Diabetes Mellitus"[Title/Abstract]

#50 "Diabetes Mellitus, Juvenile-Onset"[Title/Abstract]

#51 "Diabetes Mellitus, Juvenile Onset"[Title/Abstract]

#52 "Juvenile-Onset Diabetes Mellitus"[Title/Abstract]

#53 "IDDM"[Title/Abstract]

#54 "Juvenile-Onset Diabetes"[Title/Abstract]

#55 "Diabetes, Juvenile-Onset"[Title/Abstract]

#56 "Juvenile Onset Diabetes"[Title/Abstract]

#57 "Diabetes Mellitus, Sudden-Onset"[Title/Abstract]

#58 "Diabetes Mellitus, Sudden Onset"[Title/Abstract]

#59 "Sudden-Onset Diabetes Mellitus"[Title/Abstract]

#60 "Type 1 Diabetes Mellitus"[Title/Abstract]

#61 "Diabetes Mellitus, Insulin-Dependent, 1"[Title/Abstract]

#62 "Insulin-Dependent Diabetes Mellitus 1"[Title/Abstract]

#63 "Insulin Dependent Diabetes Mellitus 1"[Title/Abstract]

#64 "Type 1 Diabetes"[Title/Abstract]

#65 "Diabetes, Type 1"[Title/Abstract]

#66 "Diabetes Mellitus, Type I"[Title/Abstract]

#67 "Diabetes, Autoimmune"[Title/Abstract]

#68 "Autoimmune Diabetes"[Title/Abstract]

#69 "Diabetes Mellitus, Brittle"[Title/Abstract]

#70 "Brittle Diabetes Mellitus"[Title/Abstract]

#71 "Diabetes Mellitus, Ketosis-Prone"[Title/Abstract]

#72 "Diabetes Mellitus, Ketosis Prone"[Title/Abstract]

#73 "Ketosis-Prone Diabetes Mellitus"[Title/Abstract]

#74 #46 OR #47 OR #48 OR #49 OR #50 OR #51 OR #52 OR #53 OR #54 OR #55 OR #56 OR #57 OR #58 OR #59 OR #60 OR #61 OR #62 OR #63 OR #64 OR #65 OR #66 OR #67 OR #68 OR #69 OR #70 OR #71 OR #72 OR #73

#75 #11 OR #45 OR #74

#76 #10 AND #75

#77 "Randomized Controlled Trials as Topic"[Mesh]

#78 "Clinical Trials, Randomized" [Title/Abstract]

#79 "Trials, Randomized Clinical" [Title/Abstract]

#80 "Controlled Clinical Trials, Randomized" [Title/Abstract]

#81 "randomized" [Title/Abstract]

#82 "randomly" [Title/Abstract]

#83 "random？" [Title/Abstract]

#84 "Randomized Controlled Trial" [Publication Type]

#85 "placebo" [Title/Abstract]

#86 "single blind" [Title/Abstract]

#87 "double blind" [Title/Abstract]

#88 "triple blind" [Title/Abstract]

#89 #77 OR #78 OR #79 OR #80 OR #81 OR #82 OR #83 OR #84 OR #85 OR #86 OR #87 OR #88

#90 #76 AND #89

Cochrane Library：

#1 MeSH descriptor: [Amyloidosis] explode all trees

#2 ("Amyloidoses"):ti,ab,kw

#3 ("lipohypertrophy"):ti,ab,kw

#4 ("insulin-derived amyloidosis"):ti,ab,kw

#5 ("lipos"):ti,ab,kw

#6 ("subcutaneous induration"):ti,ab,kw

#7 ("subcutaneous nodules"):ti,ab,kw

#8 ("insulin lipohypertrophy"):ti,ab,kw

#9 ("endermic induration"):ti,ab,kw

#10 ("Amyloidosis, Familial"):ti,ab,kw

#11 ("Immunoglobulin Light-chain Amyloidosis"):ti,ab,kw

#12 ("Cerebral Amyloid Angiopathy, Familial"):ti,ab,kw

#13 ("Cryopyrin-Associated Periodic Syndromes"):ti,ab,kw

#14 ("Amyloid Neuropathies, Familial"):ti,ab,kw

#15 #1 OR #2 OR #3 OR #4 OR #4 OR #5 OR #6 OR #7 OR #8 OR #9 OR #10 OR #11 OR #12 OR #13 OR #14

#16 MeSH descriptor: [Diabetes Mellitus] explode all trees

#17 ("Diabetes Mellitus, Type 2"):ti,ab,kw

#18 ("Diabetes Mellitus, Type 1"):ti,ab,kw

#19 ("Diabetes Mellitus, Lipoatrophic"):ti,ab,kw

#20 ("Diabulimia"):ti,ab,kw

#21 ("Diabetes, Gestational"):ti,ab,kw

#22 ("Diabetes Complications"):ti,ab,kw

#23 ("Latent Autoimmune Diabetes in Adults"):ti,ab,kw

#24 ("Diabetes Mellitus, Experimental"):ti,ab,kw

#25 ("Donohue Syndrome"):ti,ab,kw

#26 ("Wolfram Syndrome"):ti,ab,kw

#27 ("Polyendocrinopathies, Autoimmune"):ti,ab,kw

#28 #16 OR #17 OR #18 OR #19 OR #20 OR #21 OR #22 OR #23 OR #24 OR #25 OR #26 OR #27

#29 #15 AND #28

Web of Science：

#1 TS=(" Amyloidosis " OR " Amyloidoses " OR " lipohypertrophy" OR " insulin-derived amyloidosis" OR " lipos" OR " subcutaneous induration" OR " subcutaneous nodules" OR " insulin lipohypertrophy" OR "

endermic induration ")

#2 TI=(" Amyloidosis " OR " Amyloidoses " OR " lipohypertrophy" OR " insulin-derived amyloidosis" OR " lipos" OR " subcutaneous induration" OR " subcutaneous nodules" OR " insulin lipohypertrophy" OR "

endermic induration ")

#3 AB=(" Amyloidosis " OR " Amyloidoses " OR " lipohypertrophy" OR " insulin-derived amyloidosis" OR " lipos" OR " subcutaneous induration" OR " subcutaneous nodules" OR " insulin lipohypertrophy" OR "

endermic induration ")

#4 #1 OR #2 OR #3

#5 TS=(" Diabetes Mellitus " OR " Diabetes Mellitus, Type 2 " OR " Diabetes Mellitus, Type 1" OR " Diabetes Mellitus, Insulin-Dependent " OR " Diabetes Mellitus, Insulin Dependent " OR " Insulin-Dependent Diabetes Mellitus " OR " Diabetes Mellitus, Juvenile-Onset " OR " Diabetes Mellitus, Juvenile Onset " OR " Juvenile-Onset Diabetes Mellitus " OR " IDDM " OR " Juvenile-Onset Diabetes " OR " Diabetes, Juvenile-Onset " OR " Juvenile Onset Diabetes " OR " Diabetes Mellitus, Sudden-Onset " OR " Diabetes Mellitus, Sudden Onset " OR " Sudden-Onset Diabetes Mellitus " OR " Type 1 Diabetes Mellitus " OR " Diabetes Mellitus, Insulin-Dependent, 1 " OR " Insulin-Dependent Diabetes Mellitus 1 " OR " Insulin Dependent Diabetes Mellitus 1

Type 1 Diabetes " OR " Diabetes, Type 1 " OR " Diabetes Mellitus, Type I " OR " Diabetes, Autoimmune " OR " Autoimmune Diabetes " OR " Diabetes Mellitus, Brittle " OR " Brittle Diabetes Mellitus " OR " Diabetes " OR " Mellitus, Ketosis-Prone " OR " Diabetes Mellitus, Ketosis Prone " OR " Ketosis-Prone Diabetes Mellitus " OR "Diabetes Mellitus, Noninsulin-Dependent" OR " Diabetes Mellitus, Ketosis-Resistant " OR " Diabetes" OR " Mellitus, Ketosis Resistant" OR " Ketosis-Resistant Diabetes Mellitus" OR " Diabetes Mellitus, Non Insulin Dependent" OR " Diabetes Mellitus, Non-Insulin-Dependent" OR " Non-Insulin-Dependent Diabetes Mellitus" OR " Diabetes Mellitus, Stable" OR " Stable Diabetes Mellitus" OR " Diabetes Mellitus, Type II" OR " NIDDM" OR " Diabetes Mellitus, Noninsulin Dependent" OR " Diabetes Mellitus, Maturity-Onset" OR " Diabetes Mellitus, Maturity Onset" OR " Maturity-Onset Diabetes Mellitus" OR " Maturity Onset Diabetes Mellitus" OR " MODY" OR " Diabetes Mellitus, Slow-Onset" OR " Diabetes Mellitus, Slow Onset" OR " Slow-Onset Diabetes Mellitus" OR " Type 2 Diabetes Mellitus" OR " Noninsulin-Dependent Diabetes Mellitus" OR " Noninsulin Dependent Diabetes Mellitus" OR " Maturity-Onset Diabetes" OR " Diabetes, Maturity-Onset" OR " Maturity Onset Diabetes" OR " Type 2 Diabetes" OR " Diabetes, Type 2" OR " Diabetes Mellitus, Adult-Onset" OR " Adult-Onset Diabetes Mellitus" OR " Diabetes Mellitus, Adult Onset ")

#6 TI=(" Diabetes Mellitus " OR " Diabetes Mellitus, Type 2 " OR " Diabetes Mellitus, Type 1" OR " Diabetes Mellitus, Insulin-Dependent " OR " Diabetes Mellitus, Insulin Dependent " OR " Insulin-Dependent Diabetes Mellitus " OR " Diabetes Mellitus, Juvenile-Onset " OR " Diabetes Mellitus, Juvenile Onset " OR " Juvenile-Onset Diabetes Mellitus " OR " IDDM " OR " Juvenile-Onset Diabetes " OR " Diabetes, Juvenile-Onset " OR " Juvenile Onset Diabetes " OR " Diabetes Mellitus, Sudden-Onset " OR " Diabetes Mellitus, Sudden Onset " OR " Sudden-Onset Diabetes Mellitus " OR " Type 1 Diabetes Mellitus " OR " Diabetes Mellitus, Insulin-Dependent, 1 " OR " Insulin-Dependent Diabetes Mellitus 1 " OR " Insulin Dependent Diabetes Mellitus 1

Type 1 Diabetes " OR " Diabetes, Type 1 " OR " Diabetes Mellitus, Type I " OR " Diabetes, Autoimmune " OR " Autoimmune Diabetes " OR " Diabetes Mellitus, Brittle " OR " Brittle Diabetes Mellitus " OR " Diabetes " OR " Mellitus, Ketosis-Prone " OR " Diabetes Mellitus, Ketosis Prone " OR " Ketosis-Prone Diabetes Mellitus " OR "Diabetes Mellitus, Noninsulin-Dependent" OR " Diabetes Mellitus, Ketosis-Resistant " OR " Diabetes" OR " Mellitus, Ketosis Resistant" OR " Ketosis-Resistant Diabetes Mellitus" OR " Diabetes Mellitus, Non Insulin Dependent" OR " Diabetes Mellitus, Non-Insulin-Dependent" OR " Non-Insulin-Dependent Diabetes Mellitus" OR " Diabetes Mellitus, Stable" OR " Stable Diabetes Mellitus" OR " Diabetes Mellitus, Type II" OR " NIDDM" OR " Diabetes Mellitus, Noninsulin Dependent" OR " Diabetes Mellitus, Maturity-Onset" OR " Diabetes Mellitus, Maturity Onset" OR " Maturity-Onset Diabetes Mellitus" OR " Maturity Onset Diabetes Mellitus" OR " MODY" OR " Diabetes Mellitus, Slow-Onset" OR " Diabetes Mellitus, Slow Onset" OR " Slow-Onset Diabetes Mellitus" OR " Type 2 Diabetes Mellitus" OR " Noninsulin-Dependent Diabetes Mellitus" OR " Noninsulin Dependent Diabetes Mellitus" OR " Maturity-Onset Diabetes" OR " Diabetes, Maturity-Onset" OR " Maturity Onset Diabetes" OR " Type 2 Diabetes" OR " Diabetes, Type 2" OR " Diabetes Mellitus, Adult-Onset" OR " Adult-Onset Diabetes Mellitus" OR " Diabetes Mellitus, Adult Onset ")

#7 AB=(" Diabetes Mellitus " OR " Diabetes Mellitus, Type 2 " OR " Diabetes Mellitus, Type 1" OR " Diabetes Mellitus, Insulin-Dependent " OR " Diabetes Mellitus, Insulin Dependent " OR " Insulin-Dependent Diabetes Mellitus " OR " Diabetes Mellitus, Juvenile-Onset " OR " Diabetes Mellitus, Juvenile Onset " OR " Juvenile-Onset Diabetes Mellitus " OR " IDDM " OR " Juvenile-Onset Diabetes " OR " Diabetes, Juvenile-Onset " OR " Juvenile Onset Diabetes " OR " Diabetes Mellitus, Sudden-Onset " OR " Diabetes Mellitus, Sudden Onset " OR " Sudden-Onset Diabetes Mellitus " OR " Type 1 Diabetes Mellitus " OR " Diabetes Mellitus, Insulin-Dependent, 1 " OR " Insulin-Dependent Diabetes Mellitus 1 " OR " Insulin Dependent Diabetes Mellitus 1

Type 1 Diabetes " OR " Diabetes, Type 1 " OR " Diabetes Mellitus, Type I " OR " Diabetes, Autoimmune " OR " Autoimmune Diabetes " OR " Diabetes Mellitus, Brittle " OR " Brittle Diabetes Mellitus " OR " Diabetes " OR " Mellitus, Ketosis-Prone " OR " Diabetes Mellitus, Ketosis Prone " OR " Ketosis-Prone Diabetes Mellitus " OR "Diabetes Mellitus, Noninsulin-Dependent" OR " Diabetes Mellitus, Ketosis-Resistant " OR " Diabetes" OR " Mellitus, Ketosis Resistant" OR " Ketosis-Resistant Diabetes Mellitus" OR " Diabetes Mellitus, Non Insulin Dependent" OR " Diabetes Mellitus, Non-Insulin-Dependent" OR " Non-Insulin-Dependent Diabetes Mellitus" OR " Diabetes Mellitus, Stable" OR " Stable Diabetes Mellitus" OR " Diabetes Mellitus, Type II" OR " NIDDM" OR " Diabetes Mellitus, Noninsulin Dependent" OR " Diabetes Mellitus, Maturity-Onset" OR " Diabetes Mellitus, Maturity Onset" OR " Maturity-Onset Diabetes Mellitus" OR " Maturity Onset Diabetes Mellitus" OR " MODY" OR " Diabetes Mellitus, Slow-Onset" OR " Diabetes Mellitus, Slow Onset" OR " Slow-Onset Diabetes Mellitus" OR " Type 2 Diabetes Mellitus" OR " Noninsulin-Dependent Diabetes Mellitus" OR " Noninsulin Dependent Diabetes Mellitus" OR " Maturity-Onset Diabetes" OR " Diabetes, Maturity-Onset" OR " Maturity Onset Diabetes" OR " Type 2 Diabetes" OR " Diabetes, Type 2" OR " Diabetes Mellitus, Adult-Onset" OR " Adult-Onset Diabetes Mellitus" OR " Diabetes Mellitus, Adult Onset ")

#8 #5 OR #6 OR #7

#9 #4 AND #8

Chinese National Knowledge Infrastructure (CNKI)：

( SU %= '皮下硬结' OR SU %= '皮下脂肪增生' OR SU %= '胰岛素脂肪' OR SU %= '皮下结节') AND SU %= '糖尿病' Filters: Randomized Controlled Trial

WangFang：

(主题:(皮下硬结) or 主题:(皮下脂肪增生) or 主题:(胰岛素脂肪) or 主题:(皮下结节)) and 主题:(糖尿病)

Chongqing VIP (VIP) databases：

(U=(皮下硬结 OR 皮下脂肪增生 OR 皮下结节)) AND U=糖尿病
