# Supplementary material for: Efficacy of Appropriate Techniques of Traditional Chinese Medicine and Physical Therapy in Patients With Insulin‐Associated Lipohypertrophy: A Systematic Review and Meta‐Analysis
Source: Int J Endocrinol. 2026 Aug 27;2026:5589949. doi: 10.1155/ije/5589949 (PMC13520793; doi:10.1155/ije/5589949)
Supplement: Supplementary file 1 [file IJE-2026-5589949-s001.zip › Supplemental tables.docx]

Supplemental table 1 The overall certainty of each outcome.

| Outcome | Number of studies | Included study types | Risk of bias | Inconsistency | Indirectness | Imprecision | Publication bias | Effect size (OR or proportion  [95%CI]) | GRADE |
| --- | --- | --- | --- | --- | --- | --- | --- | --- | --- |
| Curative effect | 27 | RCTs | some concerns | No serious inconsistency | No serious indirectness | No serious imprecision | No serious publication bias | 5.52 (3.98 to 7.65) | Moderate |
| HbA1c | 5 | RCTs | some concerns | No serious inconsistency | No serious indirectness | No serious imprecision | No serious publication bias | -0.42 (-0.63 to -0.2) | Moderate |
| LH diameter | 3 | RCTs | some concerns | No serious inconsistency | No serious indirectness | No serious imprecision | Potential limitations are likely due to the number of studies | -2.93 (-3.30 to -2.56) | Moderate |
| FBG | 5 | RCTs | some concerns | No serious inconsistency | No serious indirectness | No serious imprecision | Some concerns in publication bias | -0.70 (-1.41 to 0.01) | Moderate |
| 2hPG | 3 | RCTs | some concerns | No serious inconsistency | No serious indirectness | No serious imprecision | Potential limitations are likely due to the number of studies | -1.14 (-2.44 to 0.16) | Moderate |
